# Free Versus In situ Right Internal Mammary Artery as a Conduit in Coronary Artery Bypass Surgery: A Meta-Analysis

**DOI:** 10.1093/icvts/ivag062

**Published:** 2026-02-25

**Authors:** Yuta Kikuchi, Tomoki Sakata, Tomonari Shimoda, Shinichi Fukuhara, Junichi Shimamura, Makoto Hibino, Tsuyoshi Kaneko, Hiroo Takayama, Hisato Takagi, Michel Pompeu Sa, Toshiki Kuno

**Affiliations:** Department of Cardiac Surgery, Thomas Jefferson University Hospital, Philadelphia, PA 19107, United States; Department of Cardiac Surgery, Thomas Jefferson University Hospital, Philadelphia, PA 19107, United States; United States Naval Hospital Yokosuka, Yokosuka, Kanagawa 2370071, Japan; Department of Cardiac Surgery, University of Michigan, Ann Arbor, MI 48103, United States; Division of Cardiothoracic Surgery, Westchester Medical Center, Valhalla, NY 10595, United States; Division of Cardiothoracic Surgery, Westchester Medical Center, Valhalla, NY 10595, United States; Division of Cardiothoracic Surgery, Washington University School of Medicine, St. Louis, MO 63110, United States; Division of Cardiothoracic and Vascular Surgery, Columbia University Irving Medical Center, New York, NY 10032, United States; Department of Cardiovascular Surgery, Shizuoka Medical Center, Shizuoka 4110905, Japan; Department of Cardiothoracic Surgery, Heart, Vascular and Thoracic Institute, Cleveland Clinic Florida, Weston, FL 33331, United States; Cardiology Division, Massachusetts General Hospital Harvard Medical School, Boston, MA 02114, United States; Division of Cardiology, Beth Israel Deaconess Medical Center, Harvard Medical School, Boston, MA 02215, United States

**Keywords:** coronary artery bypass graft (CABG), free RIMA, in situ RIMA

## Abstract

**Objectives:**

There is debate regarding the optimal choice for a second conduit in coronary artery bypass grafting. The right internal mammary artery (RIMA) is commonly employed as a second conduit; however, it is unclear whether the free (fRIMA) or in situ (isRIMA) configuration yields superior outcomes. We performed a systematic review and meta-analysis to compare clinical outcomes between fRIMA and isRIMA as the second conduit.

**Methods:**

A comprehensive search of PubMed (MEDLINE), EMBASE, and CENTRAL was performed through May 2025 to identify studies comparing outcomes in patients undergoing coronary artery bypass grafting with either fRIMA or isRIMA as a second conduit. The outcomes of interest were overall mortality, graft occlusion, major adverse cardiac events (MACE), and repeat revascularization. Data with 95% confidence intervals (CIs) were extracted. Pooled analysis was performed using a random-effects model.

**Results:**

A total of 13 studies with 9899 patients were included (fRIMA, *n* = 3095; isRIMA, *n* = 6804). The median study follow-up duration ranged from 1 to 20 years across the studies. No statistically significant differences were observed in overall mortality (hazard ratio [95% CI] = 1.16 [0.79-1.69]), graft occlusion (1.04 [0.90-1.21]), MACE (0.87 [0.62-1.21]), and repeat revascularization (1.34 [0.68-2.66]).

**Conclusions:**

In this meta-analysis, no statistically significant differences were observed between fRIMA and isRIMA configurations across the evaluated clinical outcomes. These findings suggest that, within the limitations of available evidence, the choice between fRIMA and isRIMA may be guided by clinical context, surgeon preference, patient anatomy, and target vessel characteristics rather than expected differences in major clinical outcomes.

## Graphical abstract

**Figure:**
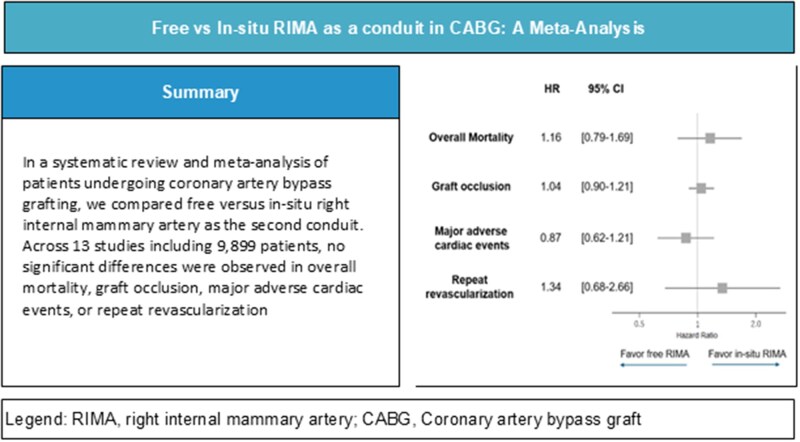

## INTRODUCTION

Coronary artery bypass grafting (CABG) is the standard of care for patients with multivessel coronary artery disease. When the left anterior descending coronary artery (LAD) is significantly stenosed, the use of the left internal mammary artery (LIMA) to LAD bypass remains the gold standard due to its superior long-term patency. However, the optimal choice of the second conduit remains a topic of ongoing debate. Commonly considered options include saphenous vein graft (SVG), radial artery, right internal mammary artery (RIMA), and gastroepiploic artery.^1^

In a comprehensive meta-analysis, Yi et al^2^ reported that the use of bilateral internal mammary arteries (IMA) was associated with improved clinical outcomes in patients with reduced left ventricular function among those with diabetes. Additionally, benefits were observed in patients across all ages,^2^^,^^3^ although the increased risk of deep sternal wound infections remains a significant concern. The superior outcomes associated with IMA grafts are largely attributed to their favourable histological characteristics, including resistance to atherosclerosis and a lower propensity for vasospasm.^4–8^

However, several studies have reported that these advantages might have been diminished when IMA is used as a free graft rather than in situ,^9–11^ potentially impacting graft patency and overall clinical outcomes. Nevertheless, evidence comparing in situ (isRIMA) versus free RIMA (fRIMA) grafting remains inconsistent. Marzouk et al^12^ reported improved long-term survival with isRIMA compared to fRIMA, while Hayashi et al^13^ found that fRIMA used for the left circumflex coronary artery (LCX) demonstrated superior long-term outcomes and significantly better patency than isRIMA.

Thus, previous studies have compared fRIMA versus isRIMA; however, most were limited by smaller sample sizes, inclusion of older studies, and heterogeneous outcome definitions and did not consistently synthesize time-to-event estimates. Therefore, uncertainty remains regarding the comparative clinical outcomes of these 2 grafting strategies.

Accordingly, we aimed to systematically evaluate and compare the clinical outcomes of fRIMA versus isRIMA grafting using a meta-analytic approach.

## METHODS

A meta-analysis was conducted in accordance with the Preferred Reporting Items for Systematic Reviews and Meta-Analyses (PRISMA) guidelines.^14^ Studies reporting clinical outcomes in patients who underwent CABG with RIMA grafting either as a free or in situ graft were identified through a two-level strategy. First, a comprehensive literature search was conducted. Electronic searches were performed in PubMed (MEDLINE), EMBASE, and Cochrane Central Register of Controlled Trials (CENTRAL) from database inception to May 1, 2025. The search strategy combined controlled vocabulary terms (Medical Subject Headings [MeSH] and EMTREE terms) with free-text keywords. Boolean operators (AND, OR) and truncation were used to maximize sensitivity (**Table S1**). Second, additional relevant studies were identified via manual screening of reference lists from the initially retrieved articles, reviews, and commentaries. All references were imported into a citation manager for consolidation, removal of duplicates, and subsequent analysis.

Eligible studies met the following criteria: peer-reviewed publications with randomized controlled trial (RCT) or observational designs (with or without statistical adjustment, including propensity score matching [PSM] or multivariate adjustment), and reporting at least 1 outcome of interest. The primary outcome is overall mortality, and the secondary outcomes include RIMA graft occlusion, major adverse cardiac events (MACE), and repeat revascularization.

Overall mortality was defined as death from any cause during follow-up. RIMA graft occlusion was defined as angiographically or CT-confirmed occlusion or severe stenosis of RIMA. MACE was defined according to the original study definitions and generally included a composite of death, myocardial infarction, and readmission due to heart failure (**Table S2**). Repeat revascularization was defined as any subsequent coronary revascularization procedure including percutaneous coronary intervention or repeat CABG.

Study quality was independently assessed by 2 investigators using Risk of Bias 2 (ROB2) for RCT and Risk of Bias in Non-randomized Studies of Intervention (ROBINS-I) for observational studies (by T.S. and Y.K.).^15^^,^^16^

From each study, hazard ratios (HRs) for overall mortality, graft occlusion, MACE, and repeat revascularization were extracted. When odds ratios (ORs) were reported instead of HRs, particularly for graft occlusion and repeat revascularization in this study, these were converted to relative risks (RRs) using the following formula, where Pref is the incidence of the outcome in the reference group.^17^ Adjusted HRs and ORs were used when available.


$$\mathrm{RR}=\frac{\mathrm{OR}}{\left(1-\mathrm{Pref})+(\mathrm{Pref}*\mathrm{OR}\right)}$$

For studies that did not report HRs directly, Kaplan-Meier curves were digitized using WebPlotDigitizer (Rohatgi A. *WebPlotDigitizer*. Available at: https://automeris.io/WebPlotDigitizer. Accessed May 1, 2025), which extracts *x* and *y* coordinates using an automated algorithm. HRs were subsequently estimated using previously validated methods. These methods have been widely used and validated in time-to-event meta-analyses, demonstrating good agreement between estimated and reported HRs when original data are available.^18–21^

A meta-analysis was performed using the “meta” package (version 8.0-2; R Foundation for Statistical Computing). HRs were synthesized using the generic inverse variance method, and pooled analyses were performed with a random-effects model. Heterogeneity among studies was assessed using the *I*^2^ and Cochran’s Q statistics. The *I*^2^ statistic quantifies the proportion of total variability across studies that is due to heterogeneity rather than chance. Cochran’s *Q* test evaluates whether observed differences in results are compatible with chance alone. A *P*-value <.05 or an *I*^2^ > 50% was considered indicative of significant heterogeneity. For each outcome, analyses were stratified according to whether statistical adjustment was applied, and subgroup-specific estimates were calculated alongside overall effects. Effect estimates were categorized as adjusted or unadjusted. Adjusted estimates included covariate-balanced HRs derived from propensity score–matched Kaplan-Meier analyses, HRs from multivariable Cox regression models, HRs from RCTs (adjusted by design), and RRs converted from adjusted ORs when available. Unadjusted estimates comprised HRs reconstructed from crude Kaplan-Meier curves or reported without multivariable adjustment.

The weighted mean follow-up was calculated using the sample size as weights. The mean follow-up durations were extracted directly when reported; when only the median follow-up was available, the median was treated as an approximation of the mean for this descriptive analysis. Studies reporting only maximum or unspecified follow-up duration were excluded from these calculations.

Sensitivity analyses were conducted by excluding studies requiring OR-to-RR conversion. To further evaluate the impact of between-study heterogeneity, heterogeneity-adjusted sensitivity analyses were also performed for all outcomes, including Baujat plot–based identification of heterogeneity-driving studies and leave-one-out analyses. In addition, subgroup analyses excluding older studies published before 2000 were performed to assess the potential impact of temporal changes in surgical techniques and perioperative management. Fixed-effect models were also evaluated.

A meta-regression analyses were conducted using Prometa 3 (Internovi, Cesena, Italy) with the following moderators: age, gender (female), chronic obstructive pulmonary disease, diabetes mellitus, ejection fraction, hyperlipidemia, hypertension, cerebral vascular disease, peripheral vascular disease, smoking history, previous myocardial infarction, and smoking status.

Publication bias was assessed using funnel plots for all outcomes. Egger’s regression test^22^ for funnel plot asymmetry was performed for overall mortality, whereas formal asymmetry tests were not applied to other outcomes because fewer than 10 studies were available per endpoint, consistent with methodological recommendations.^23^

This study is registered with PROSPERO in the National Institute for Health and Care Research. The study name is “Free Right Internal Mammary Artery vs In-situ Right Internal Mammary Artery as a Second Conduit for Coronary Artery Bypass Surgery: A Meta-Analysis” and the study ID is “1052915”.

No generative AI tools were used in the design, analysis, or writing of this manuscript.

## RESULTS

**Figure 1.**
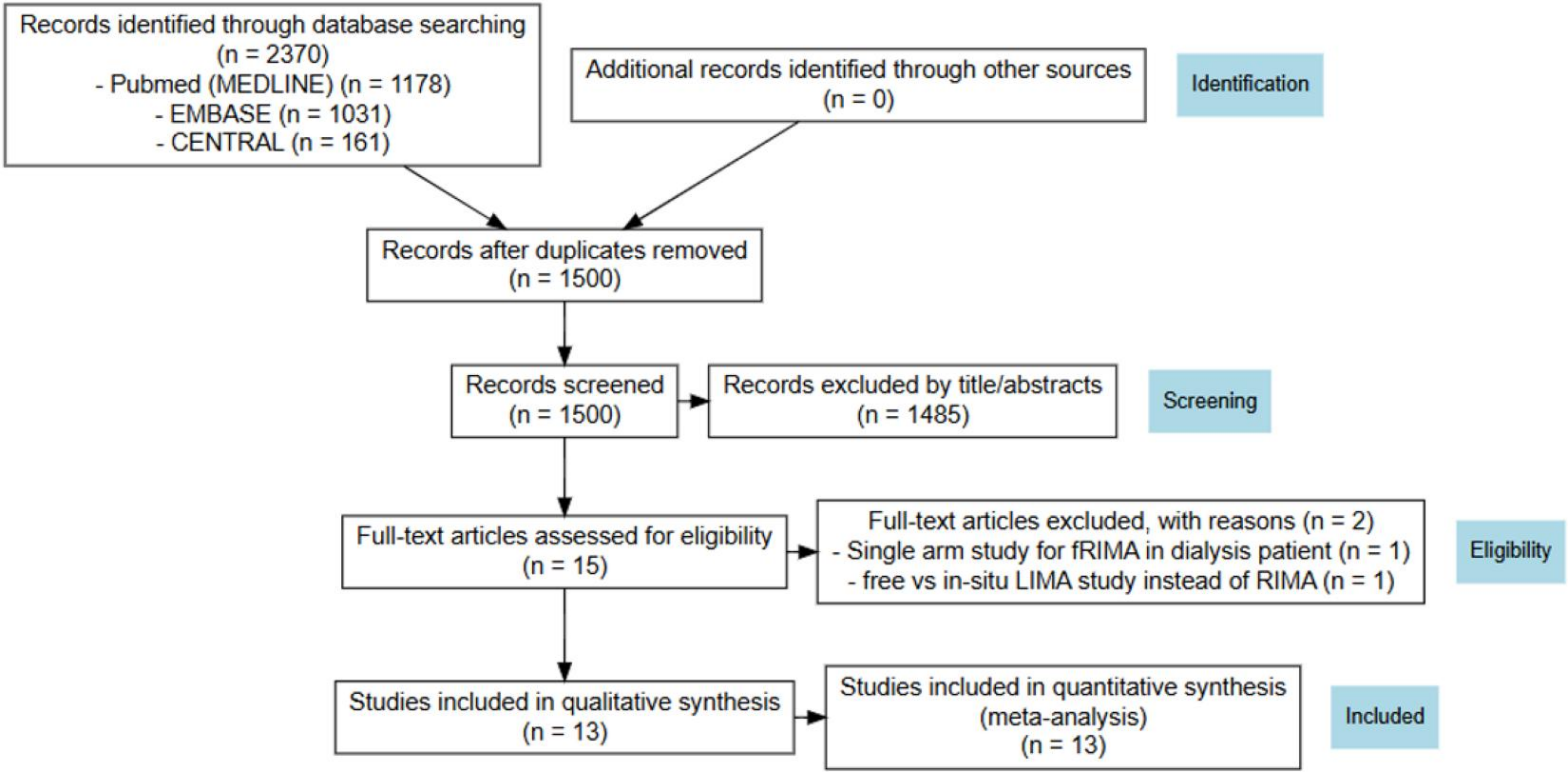
Preferred Reporting Items for Systematic Reviews and Meta-Analyses (PRISMA) Flow Diagram. Abbreviations: fRIMA, free right internal mammary artery; LIMA, left internal mammary artery.

A systematic literature search identified 1 RCT,^24^ 4 PSM studies,^12^^,^^13^^,^^25^^,^^26^ 2 studies reporting multivariable adjusted estimates,^27^^,^^28^ and 6 observational studies without statistical adjustment,^9^^,^^29^^,^^30^^,^^31–33^ encompassing a total of 9899 patients who underwent CABG with either fRIMA (*n* = 3095) or isRIMA (*n* = 6804), both in conjunction with LIMA grafting (**Figure 1**). Details of bypass target vessels, lesion severity, and RIMA graft configuration are provided in **Table S3**. RoB-2 for RCT and ROBINS-I for observational studies are summarized in **Figure S1A and B**, and no overlapping cohorts were identified among the 13 included studies (**Table S4**). The absolute event rates for all outcomes are presented in **Table S5**.

The weighted mean follow-up duration varied across outcomes (**Table S6**) and was consistently longer in the isRIMA group, largely owing to the inclusion of a single large study with extended follow-up (Marzouk et al., 2021). After exclusion of this study, weighted mean follow-up durations were comparable between groups.

The characteristics of the included studies are summarized in **Table 1**. The baseline patient demographics and clinical characteristics are detailed in **Table 2**.

**Table 1.** The Characteristics of the Included Studies

Study #	Author	Year	Design (or adjustment)	Maximum follow-up (year)	Patients (*N*)
1	Hayashi et al.	2025	PSM	10	506
2	Bakaeen et al.	2022	Multivariate	10	1331
3	Aranda-Michel et al.	2021	PSM	15	667
4	Isomura et al.	2021	NA	7	163
5	Marzouk et al.	2021	PSM	20	2493
6	Magruder et al.	2016	NA	10	577
7	Yoshizumi et al.	2012	Multivariate	10	214
8	Hwang et al.	2011	PSM	12	220
9	Tatoulis et al.	2011	NA	15	991
10	Fukui et al.	2010	NA	1	705
11	Glineur et al.	2008	RCT	3.3	304
12	Calafiore et al.	2000	NA	8	1818
13	Tashiro et al.	1998	NA	8	322

Study #	Free RIMA (N)					In situ RIMA (*N*)
	Aorta inflow	SVG inflow	LIMA inflow	Radial artery inflow	Total	
1	228	0	0	0	228	278
2	311	76	98	11	496	835
3	152	0	270	0	422	245
4	0	101	0	0	101	62
5	40	58	33	3	134	2359
6	0	99		0	99	478
7	158	0	0	0	158	56
8	0	0	110	0	110	110
9	541	0	0	0	541	450
10	158				158	547
11	0	0	152	0	152	152
12	0	0	440	0	440	1378
13	56	0	0	0	56	266

Abbreviations: LIMA, left internal mammary artery composite; N/A, not applicable; PSM, propensity score matching; RCT, randomized controlled study; RIMA, right internal mammary artery; SVG, saphenous vein graft composite.

**Table 2.** The Baseline Patient Demographics and Clinical Characteristics

Study #	Author	Age (years old)		Female (%)		DM (%)		HLD (%)	
		fRIMA	isRIMA	fRIMA	isRIMA	fRIMA	isRIMA	fRIMA	isRIMA
1	Hayashi et al.	66.9 ± 8.8	66.8 ± 9.0	20	12	58	58	72	69
2	Bakaeen et al (overall)	55.0 ± 8.3		8.6		14		NA	
3	Aranda-Michel et al.	62.9 ± 10.1	59.7 ± 9.7	17.3	8.6	40.8	31.4	75.8	80.8
4	Isomura et al.	60.7 ± 10.1	64.2 ± 11.1	22.8	12.9	36.6	38.7	62.4	59.7
5	Marzouk et al.	56.7 ± 9.5	56.2 ± 2.0	15.7	15.7	9	14.3	NA	NA
6	Magruder et al. (isRIMA-left coronary system)	55.0 ± 9.0	56.0 ± 8.0	9.1	9.6	36.4	12.6	87.9	79.1
6′	Magruder et al. (isRIMA-right coronary system)	NA	58.0 ± 10.0	NA	17.1	NA	14.2	NA	74.9
7	Yoshizumi et al.	66.2 ± 9.0	64.4 ± 9.9	24.1	7.1	61.4	55.4	60.8	57.1
8	Hwang et al.	62.6 ± 8.5	62.3 ± 8.5	22.7	21.8	37.3	37.3	20.9	23.6
9	Tatoulis et al. (overall)	60		12		11		NA	
10	Fukui et al.	66.7 ± 10.0	68.2 ± 9.2	22.2	16.5	57.6	45.7	60.8	61.6
11	Glineur et al.	62.0 ± 7.0	66.7 ± 10.0	9	14	20	19	71	71
12	Calafiore et al.	62.1 ± 9.5	61.5 ± 8.9	17.8	13.9	30.2	21.5	NA	NA
13	Tashiro et al.	62.7 ± 7.0	64.7 ± 9.0	21.4	29.3	28.6	30.8	35.7	28.6

Study #	Author	HTN (%)		Chronic renal disease (%)		Peripheral vascular disease (%)		Cerebral vascular disease (%)	
		fRIMA	isRIMA	fRIMA	isRIMA	fRIMA	isRIMA	fRIMA	isRIMA
1	Hayashi et al.	76	78	45	42	9	15	16	18
2	Bakaeen et al. (overall)	54		NA		NA		2.3	
3	Aranda-Michel et al.	83.9	83.3	NA	NA	21.3	15.9	17.3	11.4
4	Isomura et al.	70.3	72.6	NA	NA	5.9	9.7	6.9	9.7
5	Marzouk et al.	56.7	56.4	1.5	1.8	3.7	3.7	NA	NA
6	Magruder et al. (isRIMA-left coronary system)	78.8	65.7	NA	NA	7.1	6.3	5.1	2.9
6′	Magruder et al. (isRIMA-right coronary system)	NA	65.3	NA	NA	NA	5.9	NA	2.5
7	Yoshizumi et al.	75.3	66.1	2.5	10.7	NA	NA	15.8	17.9
8	Hwang et al.	65.5	58.2	1.8	3.6	NA	NA	12.7	10.0
9	Tatoulis et al. (overall)	45		NA		NA		NA	
10	Fukui et al.	65.8	66.7	NA	NA	12.7	8.2	15.2	11.7
11	Glineur et al.	70	69	NA	NA	7	12	NA	NA
12	Calafiore et al.	NA	NA	NA	NA	NA	NA	NA	NA
13	Tashiro et al.	46.4	52.6	NA	NA	NA	NA	NA	NA

Study #	Author	COPD (%)		Smoking (%)		EF (%)		EF < 30 % (%)	
		fRIMA	isRIMA	fRIMA	isRIMA	fRIMA	isRIMA	fRIMA	isRIMA
1	Hayashi et al.	5	2	62	62	59.5 ± 13.1	55.7 ± 12.3	2	3
2	Bakaeen et al. (overall)	NA		59		NA		NA	
3	Aranda-Michel et al.	15.6	15.1	23.5	25.3	51.5 ± 12.4	50.4 ± 12.4	NA	NA
4	Isomura et al.	NA	NA	59.4	58.1	NA	NA	NA	NA
5	Marzouk et al.	6	3.8	31.3	28.1	58.3 ± 12.2	58.8 ± 2.8	NA	NA
6	Magruder et al. (isRIMA-left coronary system)	7.1	3.8	14.1	51.5	52.2 ± 11.5	54.3 ± 9.6	NA	NA
6'	Magruder et al. (isRIMA-right coronary system)	NA	6.7	NA	42.7	NA	53.2 ± 10.3	NA	NA
7	Yoshizumi et al.	2.5	3.6	44.9	57.1	NA	NA	6.3	7.1
8	Hwang et al.	NA	NA	42.7	44.5	NA	NA	NA	NA
9	Tatoulis et al. (overall)	NA		NA		NA		NA	
10	Fukui et al.	6.3	3.3	63.3	59.8	55.7 ± 12.3	55.8 ± 11.9	NA	NA
11	Glineur et al.	NA	NA	37	42	NA	NA	3	3
12	Calafiore et al.	NA	NA	NA	NA	55.5 ± 14.6	59.0 ± 12.9	NA	NA
13	Tashiro et al.	NA	NA	NA	NA	NA	NA	NA	NA

Study #	Author	EF < 35 % (%)		EF < 40 % (%)		Previous MI (%)		Non-elective surgery (%)	
		fRIMA	isRIMA	fRIMA	isRIMA	fRIMA	isRIMA	fRIMA	isRIMA
1	Hayashi et al.	NA	NA	NA	NA	43	45	16	14
2	Bakaeen et al. (overall)	NA		NA		48		NA	
3	Aranda-Michel et al.	NA	NA	NA	NA	52.4	45.7	73.3	63.7
4	Isomura et al.	NA	NA	8.9	8.1	35.6	33.9		
5	Marzouk et al.	NA	NA	6.1	5.3	53	50.4	29.9	29.9
6	Magruder et al. (isRIMA-left coronary system)	NA	NA	NA	NA	NA	NA	48.5	37.7
6'	Magruder et al. (isRIMA-right coronary system)	NA	NA	NA	NA	NA	NA	NA	41.4
7	Yoshizumi et al.	NA	NA	NA	NA	NA	NA	8.9	5.4
8	Hwang et al.	6.4	6.4	NA	NA	NA	NA	NA	NA
9	Tatoulis et al. (overall)	NA		NA		18		NA	
10	Fukui et al.	NA	NA	NA	NA	51.3	48.1	4.4	14.8
11	Glineur et al.	NA	NA	NA	NA	37	28	NA	NA
12	Calafiore et al.	9.1	5.5	NA	NA	NA	NA	18.3	25.5
13	Tashiro et al.	NA	NA	16.1	12.8	46.4	63.2	10.7	16.9

Abbreviations: COPD, chronic obstructive pulmonary disease; DM, diabetes mellitus; fRIMA, free right internal mammary artery; HLD, hyperlipidemia; HTN, hypertension; is- RIMA, in situ right internal mammary artery; MI, myocardial infarction; N/A, not applicable.

**Figure 2.**
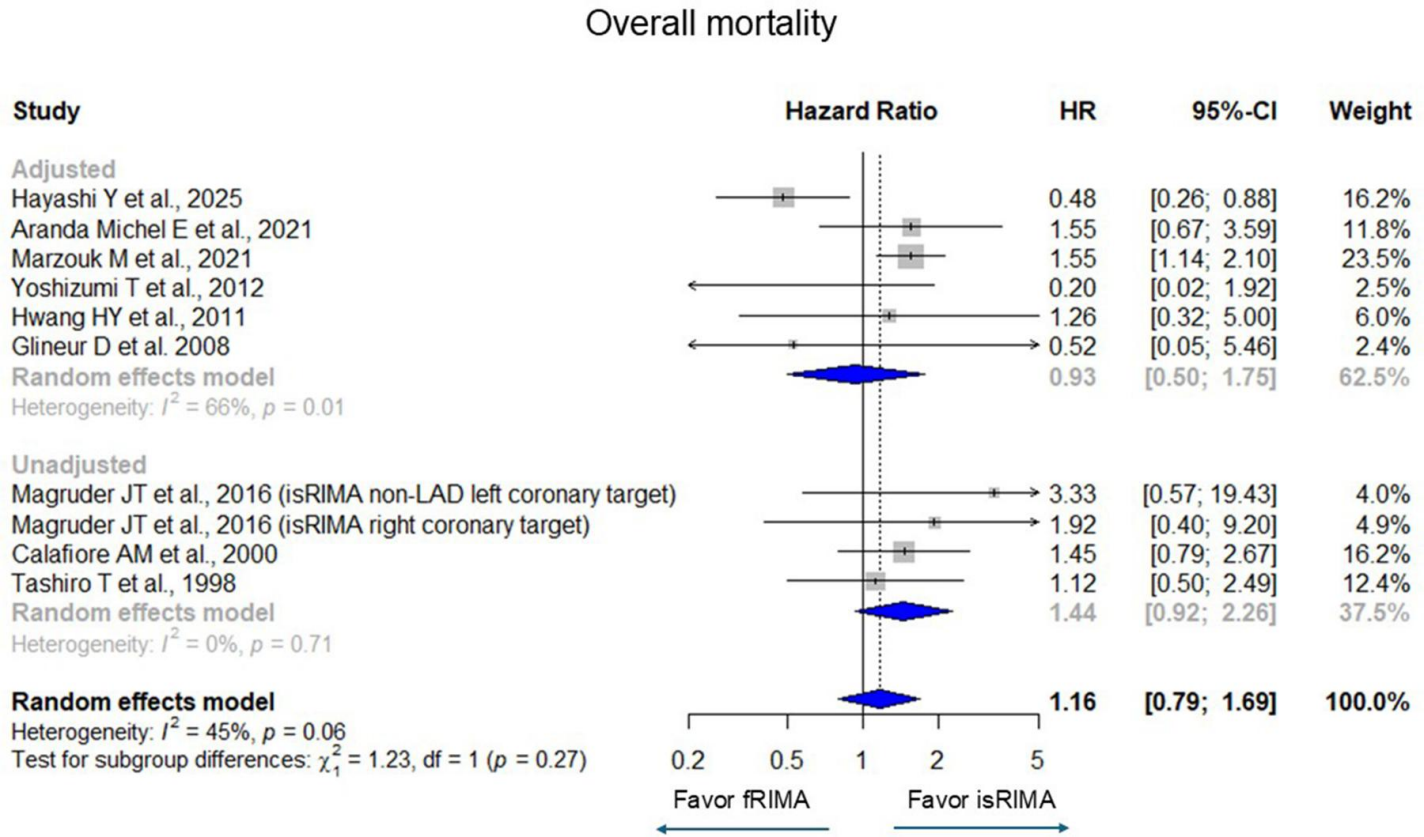
Forest Plots for Overall Mortality with Grouping Adjusted (Randomized Controlled Trial, Propensity Score Matching, and Multivariate Adjustment) and Unadjusted. The horizontal lines represent the values within the 95% CI of the underlying effects. The vertical line indicates an HR of 1. The vertical dot line indicates the mean of the total HR. Abbreviations: CI, confidence interval; HR, hazard ratio.

**Figure 3.**
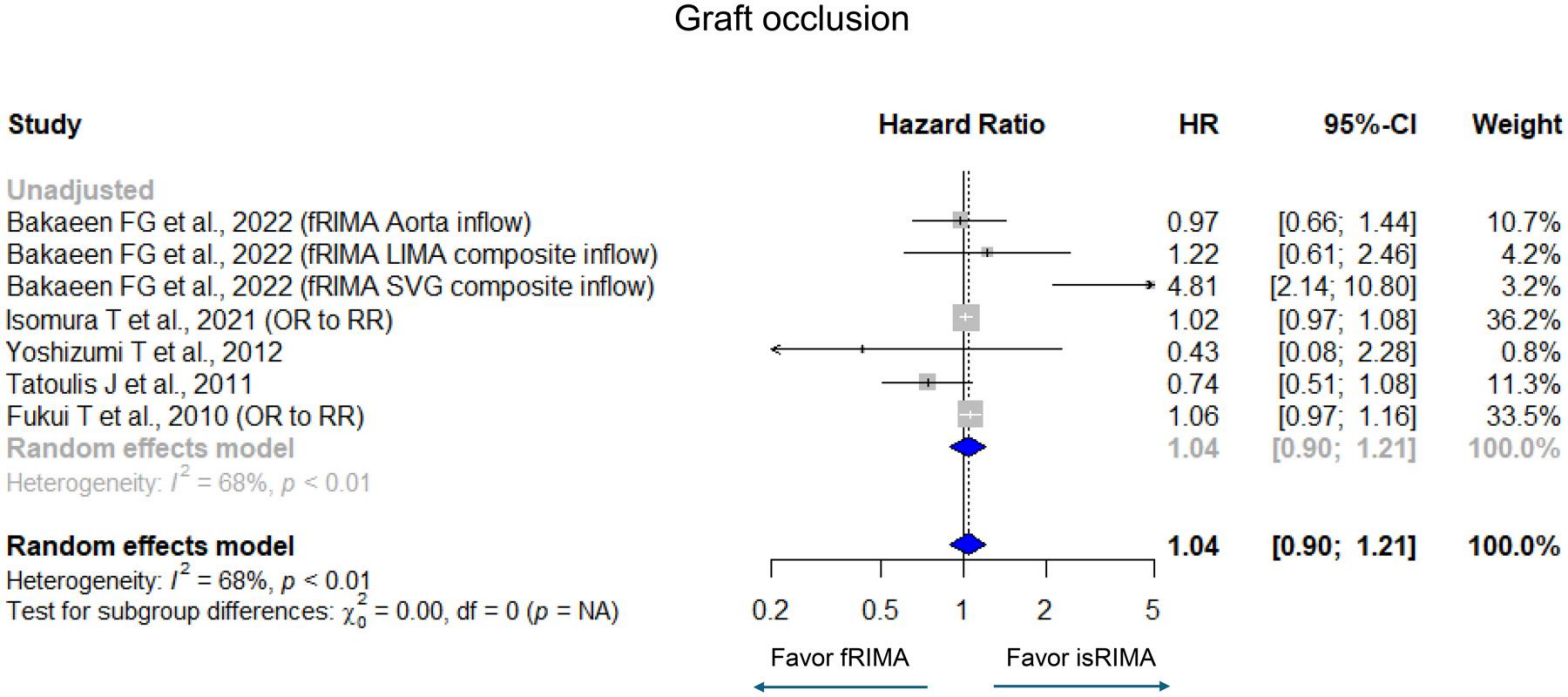
Forest Plots for Graft Occlusion with Grouping Adjusted (Randomized Controlled Trial, Propensity Score Matching, and Multivariate Adjustment) and Unadjusted. The horizontal lines represent the values within the 95% CI of the underlying effects. The vertical line indicates an HR or RR of 1. The vertical dot line indicates the mean of the total HR and RR. Abbreviations: CI, confidence interval; HR, hazard ratio; OR, odds ratio; RR, relative risk.

**Figure 4.**
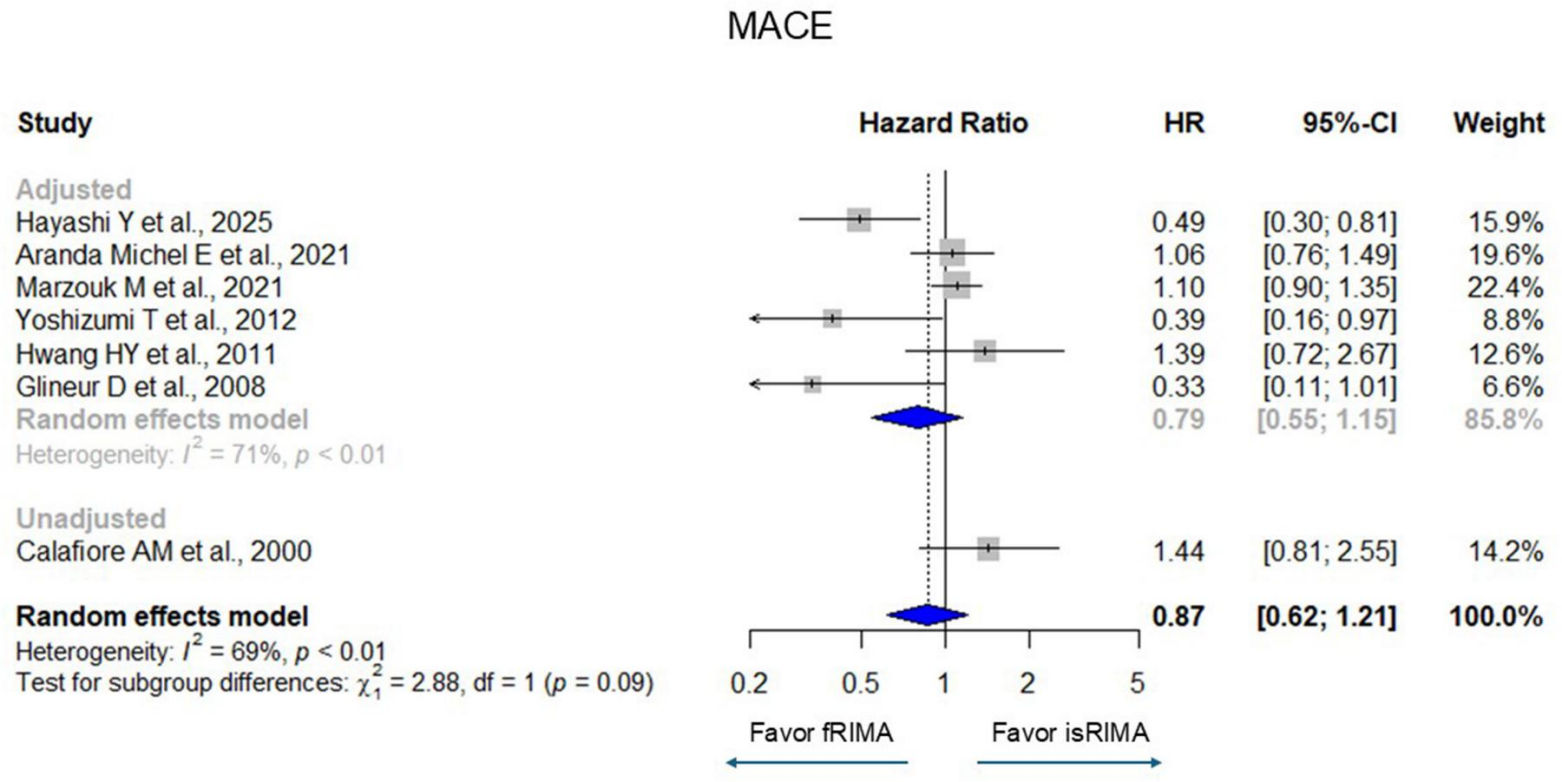
Forest Plots for Major Adverse Cardiac Event with Grouping Adjusted (Randomized Controlled Trial, Propensity Score Matching, and Multivariate Adjustment) and Unadjusted. The horizontal lines represent the values within the 95% CI of the underlying effects. The vertical line indicates an HR of 1. The vertical dot line indicates the mean of the total HR. Abbreviations: CI, confidence interval; HR, hazard ratio.

**Figure 5.**
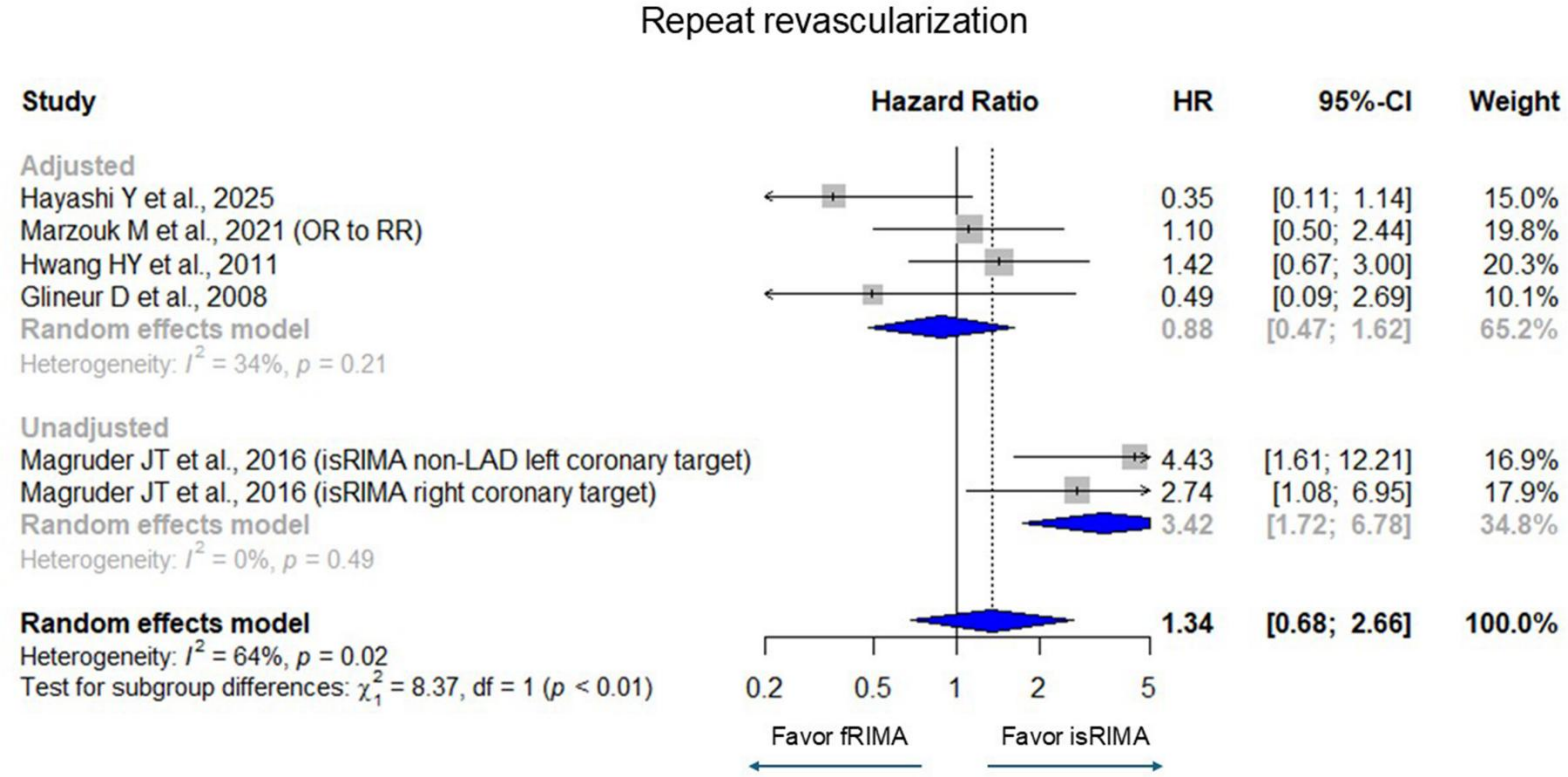
Forest Plots for Repeat Revascularization with Grouping Adjusted (Randomized Controlled Trial, Propensity Score Matching, and Multivariate Adjustment) and Unadjusted. The horizontal lines represent the values within the 95% CI of the underlying effects. The vertical line indicates an HR or RR of 1. The vertical dot line indicates the mean of the total HR and RR. Abbreviations: CI, confidence interval; HR, hazard ratio; OR, odds ratio; RR, relative risk.

Pooled analyses showed no significant difference in overall mortality between fRIMA and isRIMA (HR [95% confidence interval (CI)] = 1.16 [0.79-1.69], *I*^2^ = 45%) (**Figure 2**). Similarly, no significant differences were observed in graft occlusion (HR = 1.04 [0.90-1.21], *I*^2^ = 68%), MACE (HR = 0.87 [0.62-1.21], *I*^2^ = 69%), and repeat revascularization (HR = 1.34 [0.68-2.66], *I*^2^ = 64%) (**Figures 3-5**). There were significant heterogeneities in graft occlusion, MACE, and repeat revascularization.

Funnel plots for all outcomes are presented in **Figure S2A-D**. In the analysis of overall mortality, Egger’s regression test did not demonstrate significant funnel plot asymmetry.

The results of the sensitivity analyses excluding studies that required OR-to-RR conversion for graft occlusion and repeat revascularization are summarized in **Figure S3A and B**, and the OR-to-RR conversion value did not affect the outcomes.

The leave-one-out analyses incorporating changes in heterogeneity (*I*^2^) are summarized in **Figure S4A-D**. The pooled results were generally robust, except for overall mortality, for which a single study^13^ was identified as the primary source of heterogeneity. Exclusion of this study eliminated heterogeneity (*I*^2^ = 0%) and shifted the pooled estimate towards an isRIMA-favourable direction, although the overall interpretation remained non-significant. In contrast, the exclusion of the large study by Marzouk et al. (2021) which had extended follow-up did not materially alter the results. For all secondary outcomes, pooled estimates remained non-significant even after exclusion of heterogeneity-driving studies.

The fixed-effect models showed a statistically significant difference in overall mortality; however, this finding was not retained in the primary random-effects analysis due to between-study heterogeneity (**Figure S5A-D**). No significant differences were found in the other outcomes.

Meta-regression showed that preoperative prevalence of hypertension and peripheral vascular disease had an impact on repeat revascularization (**Figures S6A and B**). Additionally, subgroup analyses were performed excluding the studies by Tashiro et al^30^ and Calafiore et al^29^ The exclusion of these studies did not materially alter the results with no significant differences observed in overall mortality (**Figure S7A and B**).

## DISCUSSION

In this meta-analysis of 13 studies including 9899 patients, no significant differences were observed between fRIMA and isRIMA across outcomes in the primary random-effects analyses. The longer weighted mean follow-up observed in the isRIMA group was mainly attributable to a single large study and did not materially affect the overall results. Sensitivity analyses largely supported the consistency of these findings. Our findings provide an updated and robust assessment by incorporating contemporary studies, although the absence of significant differences should not be interpreted as equivalent.

Since accumulating evidence has demonstrated that total arterial revascularization is associated with improved long-term clinical outcomes, fRIMA may be considered a valid arterial conduit option when isRIMA use is anatomically or technically constrained. Although the utilization of bilateral IMA has been associated with an increased risk of sternal wound complications—particularly in diabetic patients—the use of fRIMA may still allow extension of multi- or total arterial revascularization strategies in selected cases. In the setting of off-pump CABG, where conduit length, positioning, and haemodynamic stability are critical technical considerations, the versatility of fRIMA grafting may further facilitate the achievement of multi-arterial or total arterial revascularization.

Several prospective randomized trials are underway in addition to observational evidence supporting multi-arterial grafting. The ROMA trial (NCT03217006) is a large randomized comparison of single versus multiple arterial grafting designed to assess long-term clinical outcomes and is expected to provide high-level evidence for multi-arterial strategies.^34^ Within this evolving landscape, RIMA represents a biologically favourable conduit because of its resistance to atherosclerosis and favourable endothelial function.^4–8^ However, broader adoption of fRIMA has been limited by practical considerations including increased technical complexity, longer operative time, and the lack of standardized composite graft configurations such as Y-grafts. These real-world constraints likely contribute to variability in clinical practice.

Nevertheless, our meta-analysis demonstrated that the use of isRIMA as a second conduit did not confer significant clinical advantages over fRIMA. These findings, however, should be interpreted with caution, given the variability of the targets which could impact the graft patency. Bakaeen et al^27^ demonstrated that the target coronary territory significantly influences RIMA graft patency. In their analysis, RIMA grafts to LAD had markedly superior patency compared to grafts to diagonal, LCX, or right coronary artery (RCA). This finding underscores the importance of outflow target selection in determining graft patency, whether using fRIMA or isRIMA. Additionally, Magruder et al^31^ conducted a subgroup analysis comparing LIMA-LAD and RIMA-LAD configurations. They found no significant differences in survival or the need for repeat revascularization, corroborated by several other studies.^33^^,^^35^^,^^36^ LAD remains an ideal target due to its extensive myocardial perfusion territory and consistently high graft patency regardless of conduit type.

RIMA is often employed as a second conduit to bypass LCX or RCA. When using isRIMA for LCX revascularization, conduit length can be a limiting factor. Routing isRIMA through the transverse sinus can facilitate distal LCX grafting, but may compromise patency due to risks such as aortic compression, kinking, overstretching, and twisting.^10^ Interestingly, Hayashi et al^13^ observed no significant difference in 4-year RIMA patency between the transverse sinus and non-transverse sinus routes, while they found that fRIMA to LCX may be associated with improved long-term outcomes in terms of MACE, graft patency, and all-cause mortality, compared to isRIMA configurations. The principal advantage of an fRIMA graft lies in its ability to reach and revascularize more distal segments of the LCX. Notably, no studies to date have directly compared the efficacy of isRIMA versus fRIMA specifically for RCA revascularization.

We demonstrated that we could use fRIMA as needed. However, the optimal inflow source for fRIMA remains uncertain. Commonly used inflows include the ascending aorta, a Y-composite configuration with LIMA, and the proximal hood of SVG anastomosed to the ascending aorta. The ascending aorta provides robust flow, but its use may be contraindicated in the presence of significant calcification or atheroma. A Y-composite with LIMA is advantageous as it avoids manipulation of the aorta, potentially lowering the risk of thromboembolic complications such as stroke (“no-touch” technique). In terms of graft patency, Fukui et al^9^ reported no significant difference between the ascending aorta and LIMA Y-composite inflows. To date, no studies have evaluated the impact of using SVG as an inflow for fRIMA. Furthermore, although several anastomotic techniques for fRIMA-to-aorta connections—such as the piggyback, V-composite, and foldback methods—have been described, comparative studies are lacking. Hayashi et al^36^ highlighted the efficacy of the piggyback technique relative to isRIMA for LCX bypass, but direct comparisons among various anastomotic strategies have not yet been conducted. Regardless of the conduit configuration, Bakaeen et al^13^ demonstrated that, after adjusting for target vessel location, the origin of the fRIMA did not significantly influence graft patency outcomes.

## LIMITATIONS

This study has several limitations. First, the number of included studies was modest, and substantial clinical heterogeneity existed across studies with respect to target coronary territories, severity of target lesions, RIMA configuration, and inflow sources, which were not consistently reported and may have influenced outcomes. Second, between-study heterogeneity was observed for several endpoints. Although exclusion of a single influential study eliminated heterogeneity for overall mortality and shifted the pooled estimate, the overall conclusions remained unchanged and residual heterogeneity persisted for other outcomes. Accordingly, the random-effects model was prioritized, and statistically significant findings observed only in fixed-effect analyses should be interpreted with caution. Third, when studies reported multiple effect estimates according to different inflow sources or target vessels, these were treated as independent comparisons. Fourth, some outcomes were synthesized using a combination of HRs, ORs converted to RRs and HRs reconstructed from Kaplan-Meier curves. Although prior validation studies^23^ suggest reasonable accuracy, uncertainty arising from these reconstructions was not explicitly incorporated. Fifth, although incidence rate ratios can in principle account for differences in follow-up duration, calculation of adjusted incidence rate ratios requires detailed post-matching person-time data^23^ which were not consistently reported across the included studies. As a result, adjusted incidence rate ratios could not be derived for most studies. Instead, this meta-analysis primarily relied on adjusted HRs and adjusted ORs/RRs, which were more frequently reported and allowed inclusion of a larger number of confounder-adjusted estimates. Consequently, direct comparison based on incidence rates was limited. Finally, this meta-analysis relied on aggregate study-level data, precluding patient-level adjustment, standardized time-to-event analyses, and robust assessment of effect modification; findings from meta-regression should therefore be considered exploratory.

## CONCLUSIONS

This meta-analysis found no significant differences between free and in situ RIMA in terms of overall mortality, graft occlusion, MACE, or repeat revascularization. These findings suggest that utilizing RIMA as a free graft is an acceptable and reasonable option when clinically indicated. However, variability in target vessel selection and surgical techniques across studies may have influenced the pooled outcomes, underscoring the need for further prospective research to optimize conduit strategy.

## Supplementary Material

ivag062_Supplementary_Data

## Data Availability

The data underlying this article will be shared on reasonable request to the corresponding/first author.

## ABBREVIATIONS

CABG: coronary artery bypass grafting
IMA: internal mammary artery
LAD: left anterior descending artery
LCX: left circumflex artery
LIMA: left internal mammary artery
PRISMA: preferred reporting items for systematic reviews and meta-analyses
RCA: right coronary artery
RIMA: right internal mammary artery
SVG: saphenous vein graft
