# Supplementary material for: Free Versus In situ Right Internal Mammary Artery as a Conduit in Coronary Artery Bypass Surgery: A Meta-Analysis
Source: Interdiscip Cardiovasc Thorac Surg. 2026 Feb 25;41(4):ivag062. doi: 10.1093/icvts/ivag062 (PMC13043274; doi:10.1093/icvts/ivag062)
Supplement: ivag062_Supplementary_Data [file ivag062_supplementary_data.zip › 09-Mar-2026_111732_Revised_Supplemental_Tables_ICVTS.docx]

**Supplemental Tables**

**Supplemental Table S1.**

Detailed Search Strategy.

| **Database** | **Search Date** | **Search Terms** |
| --- | --- | --- |
| PubMed (MEDLINE) | Through May 1, 2025 | (“Coronary Artery Bypass”[Mesh] OR “coronary artery bypass graft*” OR “coronary bypass*” OR CABG) AND (“Internal Mammary Artery”[Mesh] OR “internal mammary arter*” OR “internal thoracic arter*” OR RIMA OR IMA OR ITA) AND (free OR “in situ” OR in-situ) |
| EMBASE | Through May 1, 2025 | (‘coronary artery bypass’/exp OR ‘coronary artery bypass graft*’ OR CABG) AND (‘internal mammary artery’/exp OR ‘internal thoracic artery’/exp OR RIMA OR IMA OR ITA) AND (free OR ‘in situ’ OR in-situ) |
| Cochrane Central Register of Controlled Trials (CENTRAL) | Through May 1, 2025 | (coronary artery bypass OR coronary artery bypass graft* OR coronary bypass* OR CABG) AND (internal mammary artery OR internal thoracic artery OR RIMA OR IMA OR ITA) AND (free OR “in situ” OR in-situ) |
| Additional Search | - | Reference lists of all eligible articles, relevant reviews, and commentaries were manually screened to identify additional studies. Two reviewers (YK and TS) independently conducted the literature search and study selection. All records were imported into a citation management software, and duplicates were removed prior to title/abstract screening and full-text review. |

**Supplemental Table S2.**

The definitions of MACE and graft occlusion in each study. MACE, major adverse cardiac events; MI, myocardial infarction; CTA, computed tomography angiogram.

| **Study #** | **Authors** | **Definition of MACE** | **Definition of Graf occlusion** |
| --- | --- | --- | --- |
| 1 | Hayashi Y et al. | All cause mortality, MI, Revascularization | NA |
| 2 | Bakaeen FG et al. | NA | Coronary angiogram: Occlusion |
| 3 | Aranda-Michel E et al. | Repeat revasularization, MI, Stroke, Death | NA |
| 4 | Isomura T et al. | NA | Coronary CTA: Occlusion |
| 5 | Marzouk M et al. | Combined cardiovascular events, including hospital readmission for cardiac and vascular causes, and repeat revascularization | NA |
| 6 | Magruder JT et al. | NA | NA |
| 7 | Yoshizumi T et al. | Cardiac ischemia (angina pectoris recurrence, MI),  Postoperative re-revascularization, Hospitalization for heart failure, Cardiac-related death | Coronary angiogram or Coronary CTA: Occlusion or string sign |
| 8 | Hwang HY et al. | Acute MI, Coronary reintervention,  Cardiac death, including sudden death during follow-up | NA |
| 9 | Tatouli J et al. | NA | Coronary angiogram: Occlusion, string sign, or stenosis > 80% |
| 10 | Fukui T et al. | NA | Coronary angiogram: Occlusion, string sign, or stenosis > 90% |
| 11 | Glineur D et al. | Death from any cause; Perioperative MI,  Additional cardiac surgery, Coronary angioplasty, Stroke | NA |
| 12 | Calafiore AM et al. | Acute MI, Need for PCI, Need for reoperation | NA |
| 13 | Tashiro T et al. | NA | NA |

**Supplemental Table S3.**

The bypass target vessels and the severity of stenosis in the corresponding native coronary arteries in each study. fRIMA, free right internal mammary artery; isRIMA, in-situ right internal mammary artery; LAD, left anterior descending coronary artery; LCX, left circumflex coronary artery; OM, obtuse marginal; RCA, right coronary artery; N/A, not applicable.

| **Study #** | **Author** | **fRIMA** | | | | | | |
| --- | --- | --- | --- | --- | --- | --- | --- | --- |
|  |  | **Sequential (%)** | **LAD (N)** | **Diagonal (N)** | **Ramus (N)** | **LCX (N)** | **OM (N)** | **RCA (N)** |
| 1 | Hayashi Y et al. | 45.0 | NA | NA | NA | 228 | NA | NA |
| 2 | Bakaeen FG et al. | NA | 74 | 63 | NA | 327 | NA | 77 |
| 3 | Aranda-Michel E et al. | NA | 9 | 62 | 58 | 14 | 238 | 37 |
| 4 | Isomura T et al. | 85.1 | 3 | 55 | 34 | 28 | 72 | 12 |
| 5 | Marzouk M et al. | NA | | | | | | |
| 6 | Magruder JT et al. | NA | NA | 2 | 12 | NA | 82 | 3 |
| 7 | Yoshizumi T et al. | NA | | | | | | |
| 8 | Hwang HY et al. | 52.7 | NA | | | | | |
| 9 | Tatouli J et al. | NA | | | | | | |
| 10 | Fukui T et al. | NA | NA | 26 | NA | 132 | NA | NA |
| 11 | Glineur D et al. | NA | NA | NA | 14 | NA | 220 | 40 |
| 12 | Calafiore AM et al. | 34.3 | 50 | 67 | NA | 470 | | 38 |
| 13 | Tashiro T et al. | N/A | | | | | | |

| **Study #** | **Author** | **isRIMA** | | | | | | |
| --- | --- | --- | --- | --- | --- | --- | --- | --- |
|  |  | **Sequential (%)** | **LAD (N)** | **Diagonal (N)** | **Ramus (N)** | **LCX (N)** | **OM (N)** | **RCA (N)** |
| 1 | Hayashi Y et al. | 2.0 | NA | NA | NA | 278 | NA | NA |
| 2 | Bakaeen FG et al. | NA | 412 | 61 | NA | 147 | NA | 235 |
| 3 | Aranda-Michel E et al. | NA | 30 | 18 | 48 | 4 | 73 | 69 |
| 4 | Isomura T et al. | 0 | 15 | 2 | 3 | 3 | 21 | 2 |
| 5 | Marzouk M et al. | NA | | | | | | |
| 6 | Magruder JT et al. | NA | NA | 39 | 29 | 2 | 169 | 239 |
| 7 | Yoshizumi T et al. | NA | | | | | | |
| 8 | Hwang HY et al. | 41.8 | NA | | | | | |
| 9 | Tatouli J et al. | NA | | | | | | |
| 10 | Fukui T et al. | NA | 124 | 262 | NA | 128 | NA | NA |
| 11 | Glineur D et al. | NA | NA | NA | 2 | NA | 153 | 2 |
| 12 | Calafiore AM et al. | 4.1 | 998 | 58 | NA | 236 | | 242 |
| 13 | Tashiro T et al. | NA | | | | | | |

| **Study #** | **Author** | **Severity of target lesions** | |
| --- | --- | --- | --- |
|  |  | **fRIMA** | **isRIMA** |
| 1 | Hayashi Y et al. | LAD region : >75% (75%), ≤75% (25%) LCX region : >75% (65%), ≤75% (35%) | LAD region : >75% (68%), ≤75% (31%) LCX region : >75% (62%), ≤75% (38%) |
| 2 | Bakaeen FG et al. | Left main : ≥50% (18%), ≥70% (8.2%) LAD : ≥50% (95%), ≥70% (84%) LCX : ≥50% (85%), ≥70% (71%) RCA : ≥50% (89%), ≥70% (74%) | |
| 3 | Aranda-Michel E et al. | NA | |
| 4 | Isomura T et al. | NA | |
| 5 | Marzouk M et al. | NA | |
| 6 | Magruder JT et al. | NA | |
| 7 | Yoshizumi T et al. | Target coronary stenosis: 87.1±9.71% | Target coronary stenosis: 85.8±8.96% |
| 8 | Hwang HY et al. | NA | |
| 9 | Tatouli J et al. | Native coronary artery stenosis < 60% (9.7 %), 60-79% (29.2 %), 80-99% (45.3%), 100% (15.8%) | |
| 10 | Fukui T et al. | NA | |
| 11 | Glineur D et al. | >70% | |
| 12 | Calafiore AM et al. | NA | |
| 13 | Tashiro T et al. | NA | |

**Supplemental Table S4.**

Assessment of potential cohort overlap among included studies.

| **Study (First author, year)** | **Country** | **Institution / Center** | **Study design** | **Recruitment period** | **Sample size (n)** | **Potential overlap with other studies** | **Overlap** |
| --- | --- | --- | --- | --- | --- | --- | --- |
| Hayashi Y et al., 2025 | Japan | Japanese Red Cross Aichi Medical Center Nagoya Daiichi Hospital/Kurashiki Central Hospital/Kyoto Prefectural University Hospital/Toyohashi Heart Center | Retrospective | 2009-2015 | 1171 | None identified | No overlap |
| Bakaeen FG et al., 2022 | USA | Cleveland Clinic | Retrospective | 1972-2016 | 1331 | Internal subgroup only | No inter-study overlap |
| Aranda Michel E et al., 2021 | USA | University of Pittsburgh Medical Center | Retrospective | 2010-2018 | 667 | None identified | No overlap |
| Isomura T et al., 2021 | Japan | IMS Tokyo Katsushika General Hospital/Okayama University Hospital | Retrospective | 2005-2018 | 163 | None identified | No overlap |
| Marzouk M et al., 2021 | Canada | National registry | Propensity-matched | 2000–2015 | 2493 | None identified | No overlap |
| Magruder JT et al., 2016 | USA | Johns Hopkins Hospital | Retrospective | 1997–2014 | 762 | Internal subgroup only | No inter-study overlap |
| Yoshizumi T et al., 2012 | Japan | Nagoya Univ. Hospital | Retrospective | 200-2010 | 214 | None identified | No overlap |
| Hwang HY et al., 2011 | Korea | Seoul Nat’l Univ. Hospital | Propensity-matched | 1998–2003 | 220 | None identified | No overlap |
| Tatoulis J et al., 2011 | Australia | Royal Melbourne Hospital | Retrospective | 1989–2008 | 5766 | None identified | No overlap |
| Fukui T et al., 2010 | Japan | Sakakibara Heart Instute | Retrospective | 2004-2008 | 705 | None identified | No overlap |
| Glineur D et al., 2008 | Belgium | UCL, Brussels | Randomized trial | 2003–2006 | 304 | None identified | No overlap |
| Calafiore AM et al., 2000 | Italy | Univ. of Chieti | Retrospective | 1991–2000 | 1818 | None identified | No overlap |
| Tashiro T et al., 1998 | Japan | Fukuoka Univ. | Retrospective | 1988–1995 | 322 | None identified | No overlap |

**Supplemental Table S5.**

The absolute event rates for Overall mortality, Graft occlusion, MACE, and Repeat revascularization. fRIMA, free right internal mammary artery; isRIMA, in-situ right internal mammary artery; MACE; major adverse cardiac events.

| **Overall Mortality** | | | | |
| --- | --- | --- | --- | --- |
| **fRIMA** | **Study** | **n** | **Events** | **Absolute event rate (%)** |
|  | Hayashi Y et al., 2025 | 278 | 36 | 13 |
|  | Aranda Michel E et al., 2021 | 422 | 35 | 8 |
|  | Marzouk M et al., 2021 | 134 | 41 | 31 |
|  | Magruder JT et al., 2016 | 123 | 7 | 6 |
|  | Yoshizumi T et al., 2012 | 158 | 5 | 3 |
|  | Hwang HY et al., 2011 | 110 | 24 | 22 |
|  | Glineur D et al. 2008 | 152 | 1 | 1 |
|  | Calafiore AM et al., 2000 | 440 | 9 | 2 |
|  | Tashiro T et al., 1998 | 56 | 9 | 16 |
| **isRIMA** | **Study** | **n** | **Events** | **Absolute event rate (%)** |
|  | Hayashi Y et al., 2025 | 665 | 184 | 28 |
|  | Aranda Michel E et al., 2021 | 245 | 13 | 5 |
|  | Marzouk M et al., 2021 | 2359 | 500 | 21 |
|  | Magruder JT et al., 2016 (isRIMA non-LAD left coronary target) | 239 | 17 | 7 |
|  | Magruder JT et al., 2016 (isRIMA right coronary target) | 239 | 19 | 8 |
|  | Yoshizumi T et al., 2012 | 56 | 6 | 11 |
|  | Hwang HY et al., 2011 | 110 | 19 | 17 |
|  | Glineur D et al. 2008 | 147 | 2 | 1 |
|  | Calafiore AM et al., 2000 | 1378 | 33 | 2 |
|  | Tashiro T et al., 1998 | 266 | 27 | 10 |

| **Graft occlusion** | | | | |
| --- | --- | --- | --- | --- |
| **fRIMA** | **Study** | **n** | **Events** | **Absolute event rate (%)** |
|  | Bakaeen FG et al., 2022 (fRIMA Aorta inflow) | 478 | 74 | 15 |
|  | Bakaeen FG et al., 2022 (fRIMA LIMA composite inflow) | 125 | 21 | 17 |
|  | Bakaeen FG et al., 2022 (fRIMA SVG composite inflow) | 78 | 22 | 28 |
|  | Isomura T et al., 2021 (OR to RR) | 101 | 1 | 1 |
|  | Yoshizumi T et al., 2012 | 158 | 5 | 3 |
|  | Tatoulis J et al., 2011 | 541 | 49 | 9 |
|  | Fukui T et al., 2010 (OR to RR) | 59 | 6 | 10 |
| **isRIMA** | **Study** | **n** | **Events** | **Absolute event rate (%)** |
|  | Bakaeen FG et al., 2022 | 1289 | 209 | 16 |
|  | Isomura T et al., 2021 (OR to RR) | 62 | 2 | 3 |
|  | Yoshizumi T et al., 2012 | 56 | 11 | 20 |
|  | Tatoulis J et al., 2011 | 450 | 50 | 11 |
|  | Fukui T et al., 2010 (OR to RR) | 277 | 13 | 5 |

| **MACE** | | | | |
| --- | --- | --- | --- | --- |
| **fRIMA** | **Study** | **n** | **Events** | **Absolute event rate (%)** |
|  | Hayashi Y et al., 2025 | 228 | 46 | 20 |
|  | Aranda Michel E et al., 2021 | 422 | 154 | 36 |
|  | Marzouk M et al., 2021 | 134 | 33 | 25 |
|  | Yoshizumi T et al., 2012 | 158 | 16 | 10 |
|  | Hwang HY et al., 2011 | 110 | 22 | 20 |
|  | Glineur D et al., 2008 | 152 | 1 | 1 |
|  | Calafiore AM et al., 2000 | 440 | 28 | 6 |
| **isRIMA** | **Study** | **n** | **Events** | **Absolute event rate (%)** |
|  | Hayashi Y et al., 2025 | 665 | 248 | 37 |
|  | Aranda Michel E et al., 2021 | 245 | 89 | 36 |
|  | Marzouk M et al., 2021 | 2359 | 497 | 21 |
|  | Yoshizumi T et al., 2012 | 56 | 15 | 27 |
|  | Hwang HY et al., 2011 | 110 | 18 | 16 |
|  | Glineur D et al., 2008 | 147 | 6 | 4 |
|  | Calafiore AM et al., 2000 | 1378 | 66 | 5 |

| **Repeat revascularization** | | | | |
| --- | --- | --- | --- | --- |
| **fRIMA** | **Study** | **n** | **Events** | **Absolute event rate (%)** |
|  | Hayashi Y et al., 2025 | 228 | 17 | 7 |
|  | Marzouk M et al., 2021 (OR to RR) | 134 | 16 | 12 |
|  | Magruder JT et al., 2016 | 123 | 9 | 7 |
|  | Hwang HY et al., 2011 | 110 | 19 | 17 |
|  | Glineur D et al., 2008 | 152 | 2 | 1 |
| **isRIMA** | **Study** | **n** | **Events** | **Absolute event rate (%)** |
|  | Hayashi Y et al., 2025 | 665 | 100 | 15 |
|  | Marzouk M et al., 2021 (OR to RR) | 2359 | 236 | 10 |
|  | Magruder JT et al., 2016 (isRIMA non-LAD left coronary target) | 239 | 18 | 8 |
|  | Magruder JT et al., 2016 (isRIMA right coronary target) | 239 | 20 | 8 |
|  | Hwang HY et al., 2011 | 110 | 16 | 15 |
|  | Glineur D et al., 2008 | 147 | 4 | 3 |

**Supplemental Table S6.**

Outcome-specific weighted mean follow-up durations stratified by grafting strategy.

fRIMA, free right internal mammary artery; isRIMA, in-situ right internal mammary artery; MACE, major adverse cardiac events.

|  | **Weighted mean follow-up (years)** | | **Weighted mean follow-up without the study by Marzouk M et al., 2021 (years)** | |
| --- | --- | --- | --- | --- |
|  | **fRIMA** | **isRIMA** | **fRIMA** | **isRIMA** |
| **Overall mortality** | 4.4 | 7.3 | 3.6 | 4.0 |
| **Graft occlusion** | 1.8 | 1.6 | - | - |
| **MACE** | 5.0 | 7.8 | 3.8 | 3.9 |
| **Repeat revascularization** | 5.7 | 9.2 | 4.4 | 5.2 |
