# Supplementary figures and images for: Free Versus In situ Right Internal Mammary Artery as a Conduit in Coronary Artery Bypass Surgery: A Meta-Analysis
Source: Interdiscip Cardiovasc Thorac Surg. 2026 Feb 25;41(4):ivag062. doi: 10.1093/icvts/ivag062 (PMC13043274; doi:10.1093/icvts/ivag062)

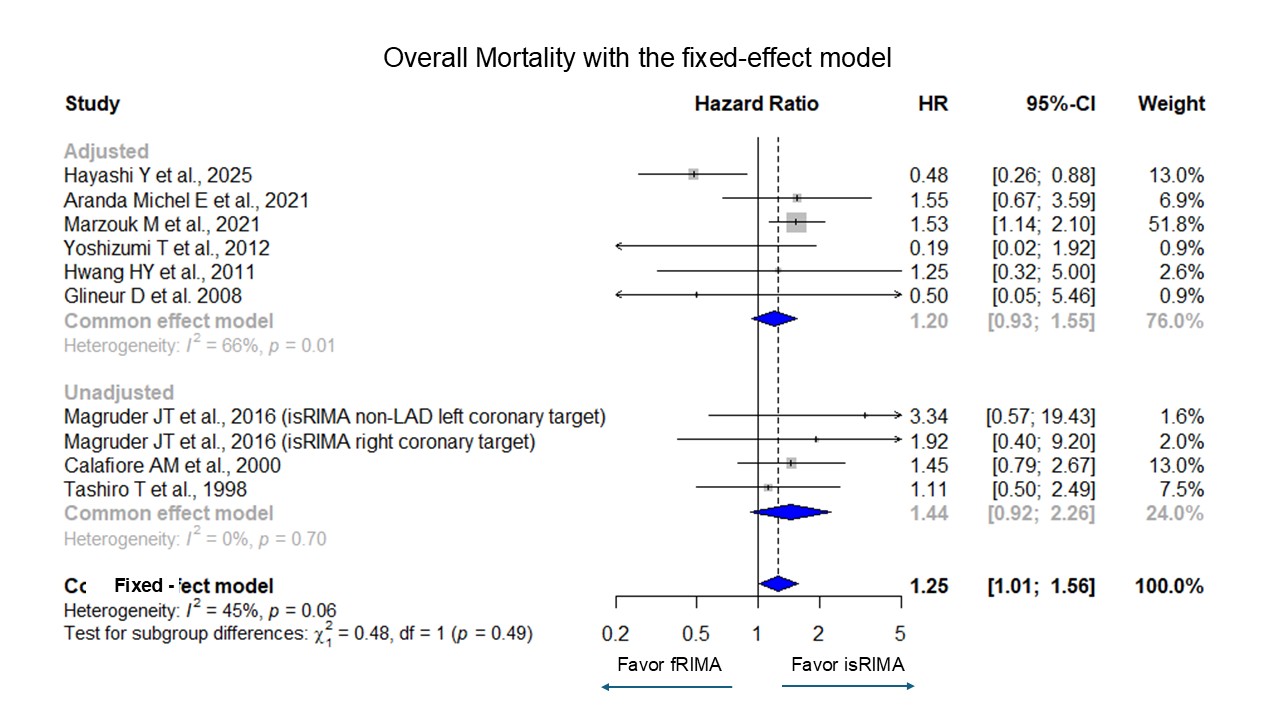

Supplement: ivag062_Supplementary_Data [file ivag062_supplementary_data.zip › 09-Mar-2026_111714_Revised_Supplemental_Figure_S5A..jpg]

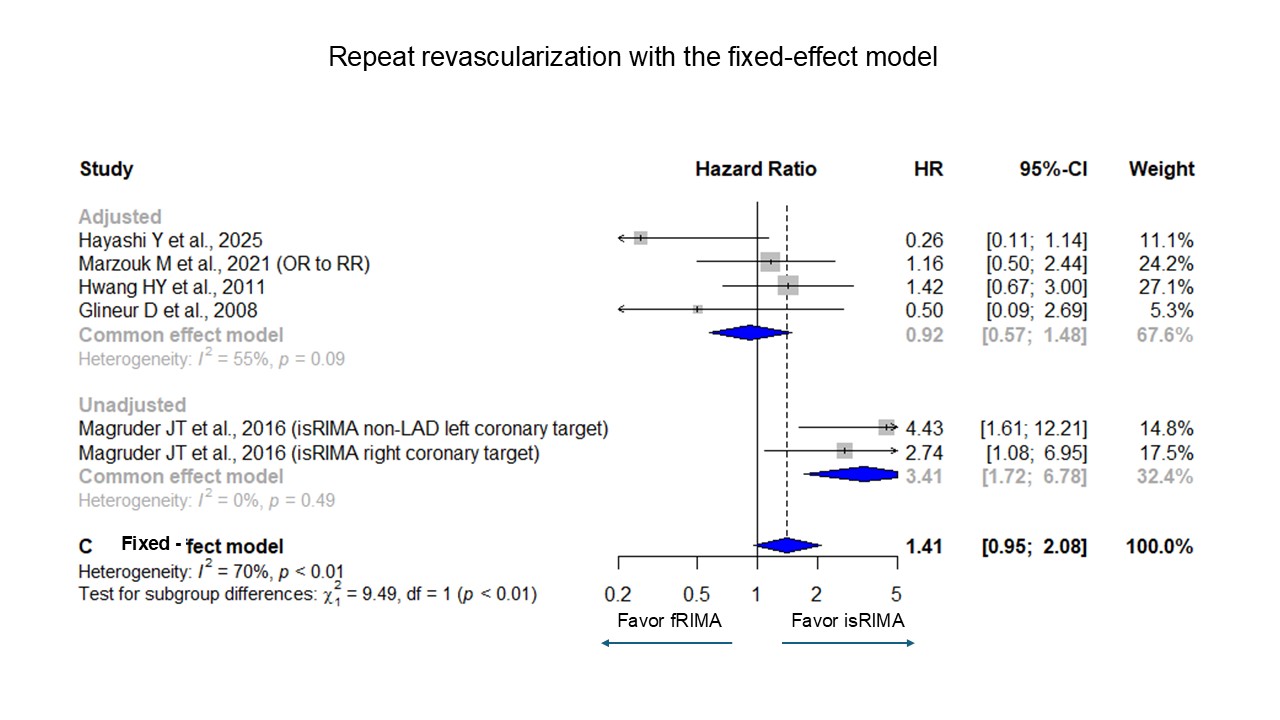

Supplement: ivag062_Supplementary_Data [file ivag062_supplementary_data.zip › 09-Mar-2026_111722_Revised_Supplemental_Figure_S5D..png.jpg]

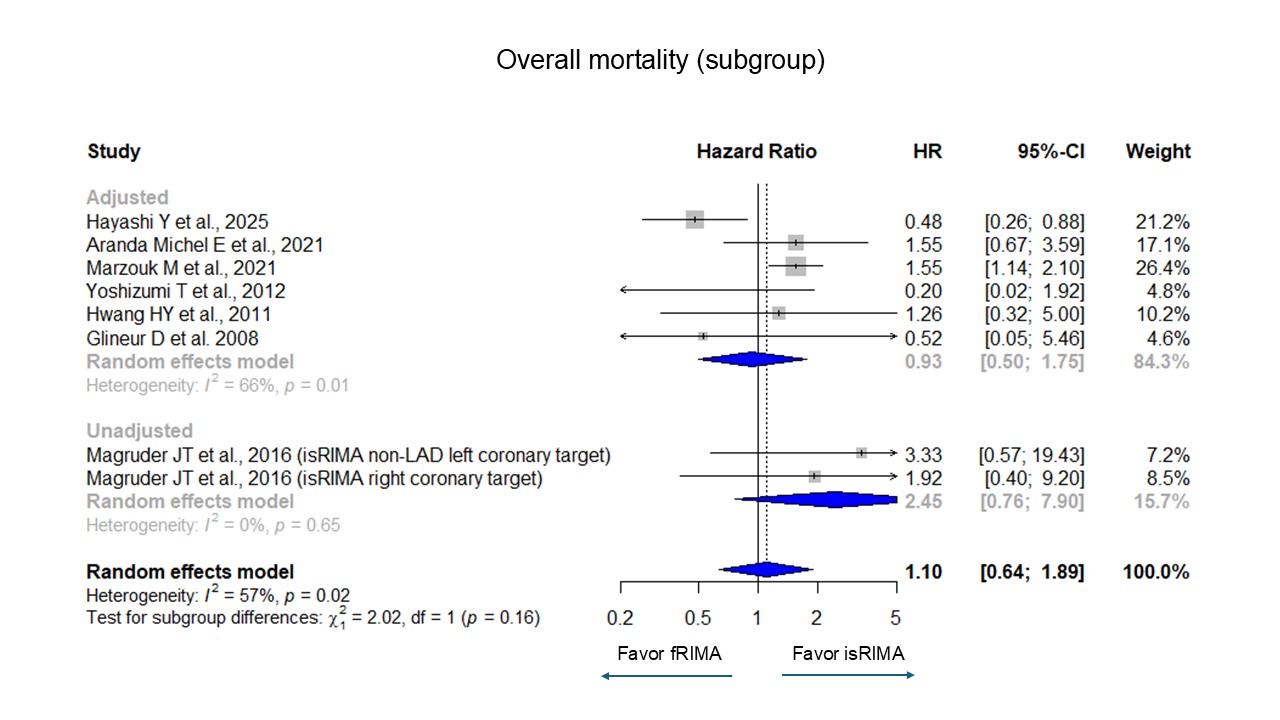

Supplement: ivag062_Supplementary_Data [file ivag062_supplementary_data.zip › 09-Mar-2026_111727_Revised_Supplemental_Figure_S7A..png.jpg]

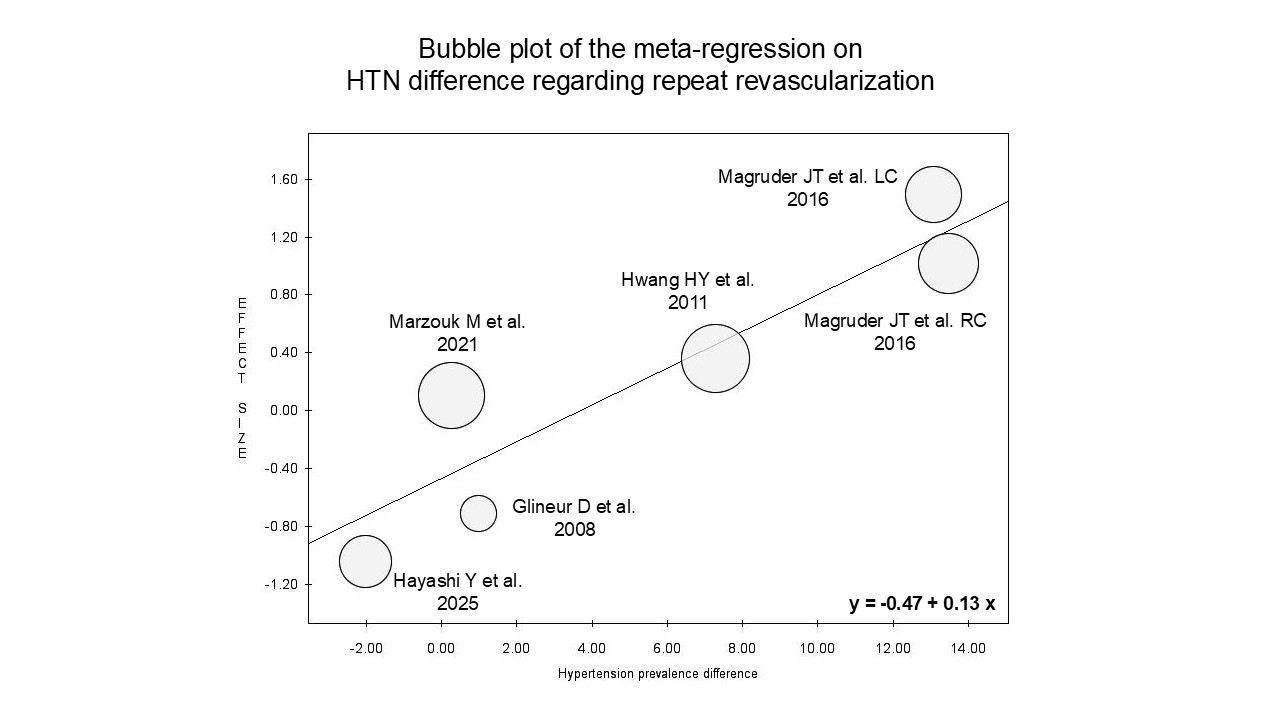

Supplement: ivag062_Supplementary_Data [file ivag062_supplementary_data.zip › Revised_Supplemental_Figure_S6A.jpg]

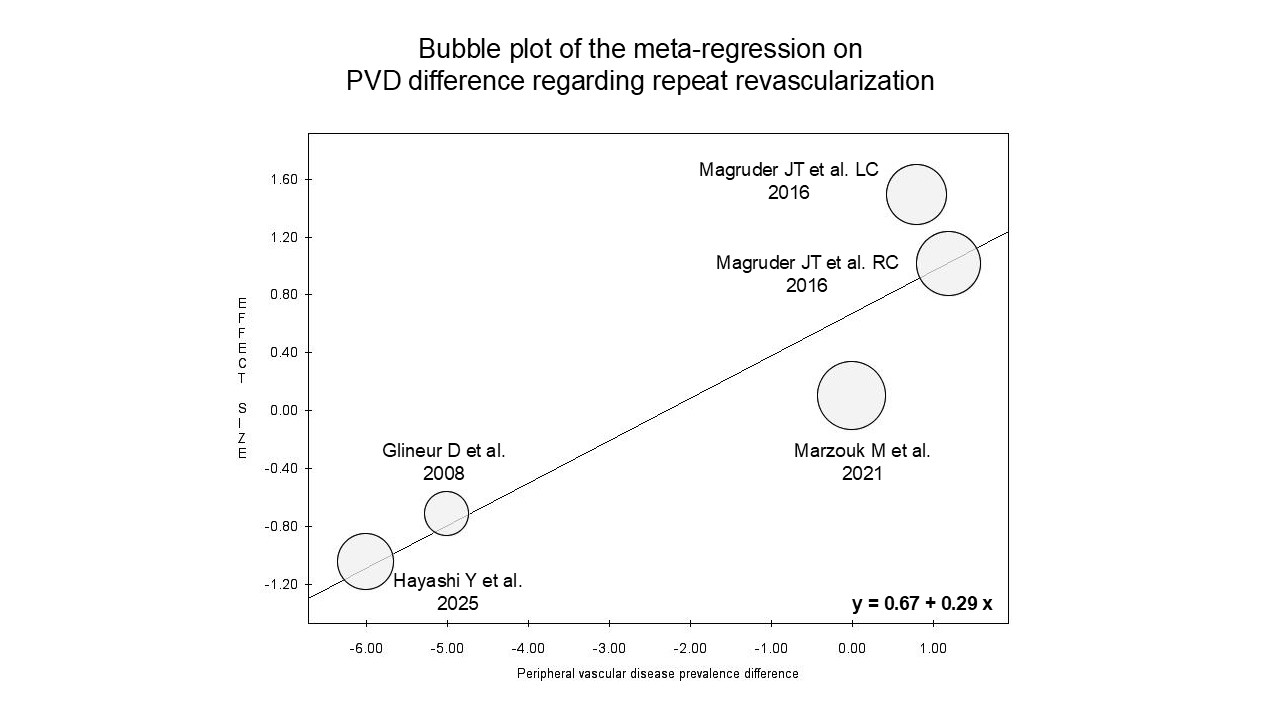

Supplement: ivag062_Supplementary_Data [file ivag062_supplementary_data.zip › 09-Mar-2026_111726_Revised_Supplemental_Figure_S6B.jpg]

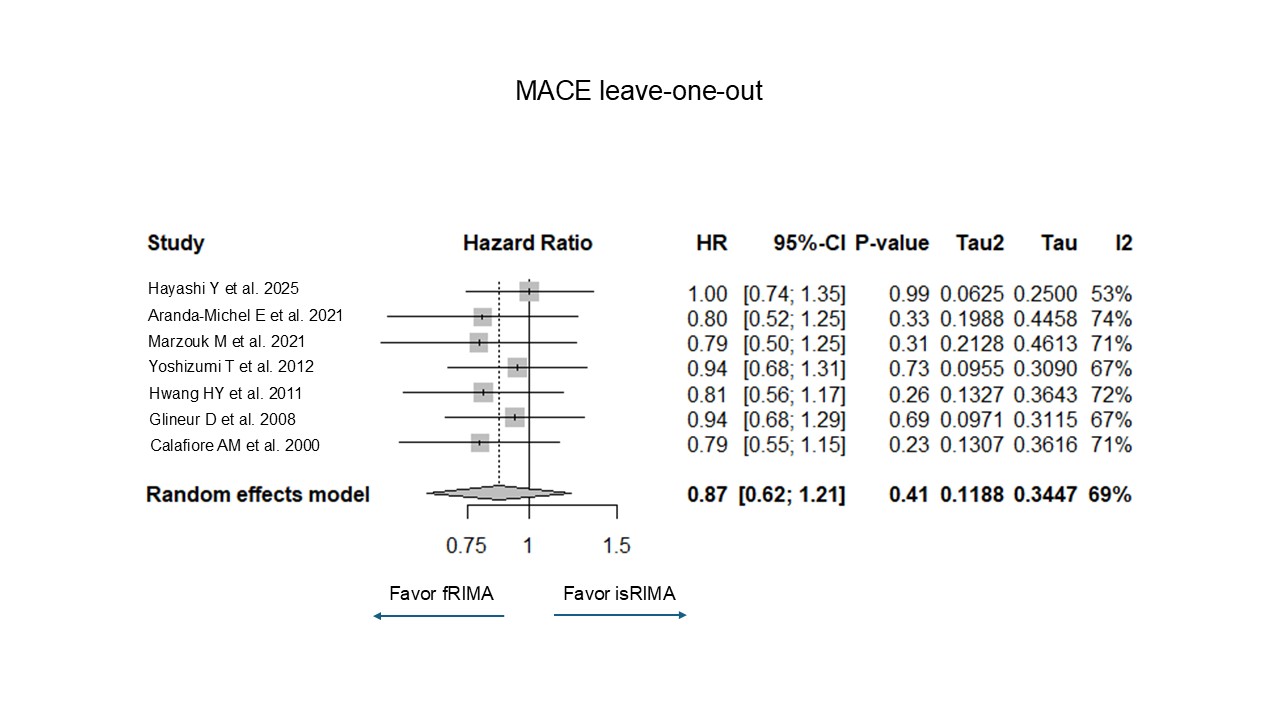

Supplement: ivag062_Supplementary_Data [file ivag062_supplementary_data.zip › Revised_Supplemental_Figure_S4C.jpg]

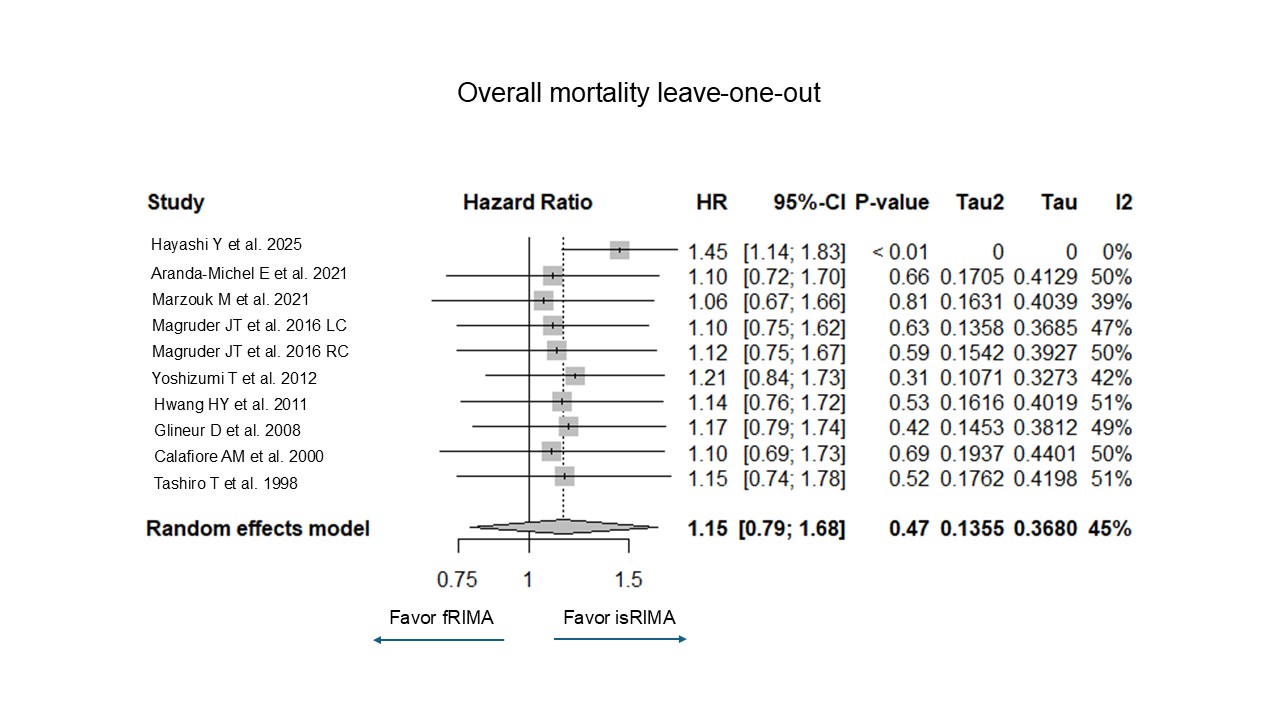

Supplement: ivag062_Supplementary_Data [file ivag062_supplementary_data.zip › 09-Mar-2026_111711_Revised_Supplemental_Figure_S4A.jpg]

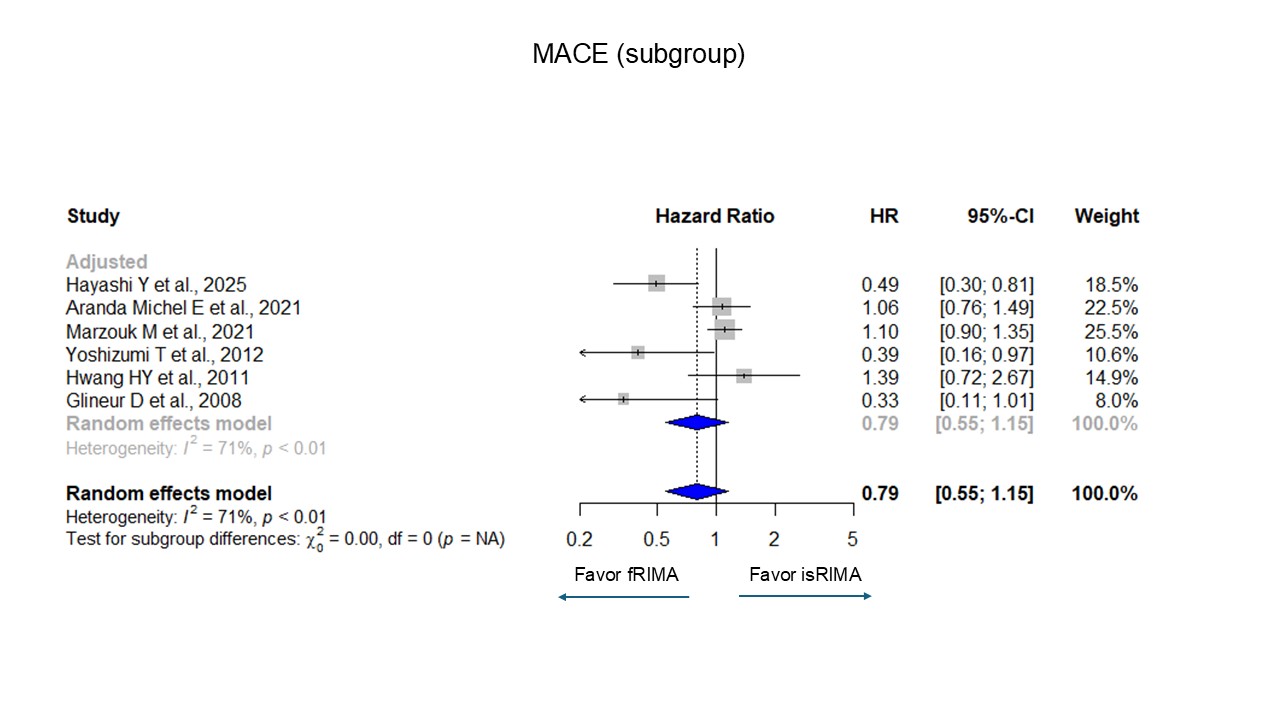

Supplement: ivag062_Supplementary_Data [file ivag062_supplementary_data.zip › Revised_Supplemental_Figure_S7B..png.jpg]

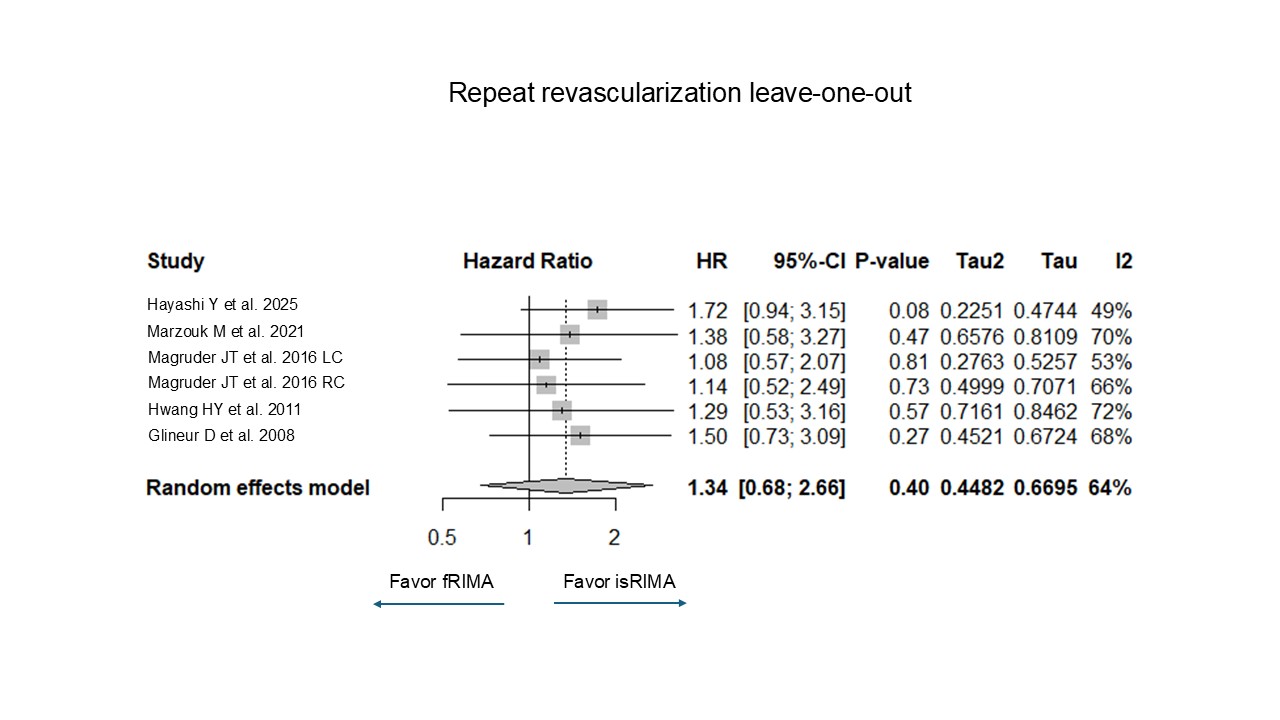

Supplement: ivag062_Supplementary_Data [file ivag062_supplementary_data.zip › 09-Mar-2026_111714_Revised_Supplemental_Figure_S4D.jpg]

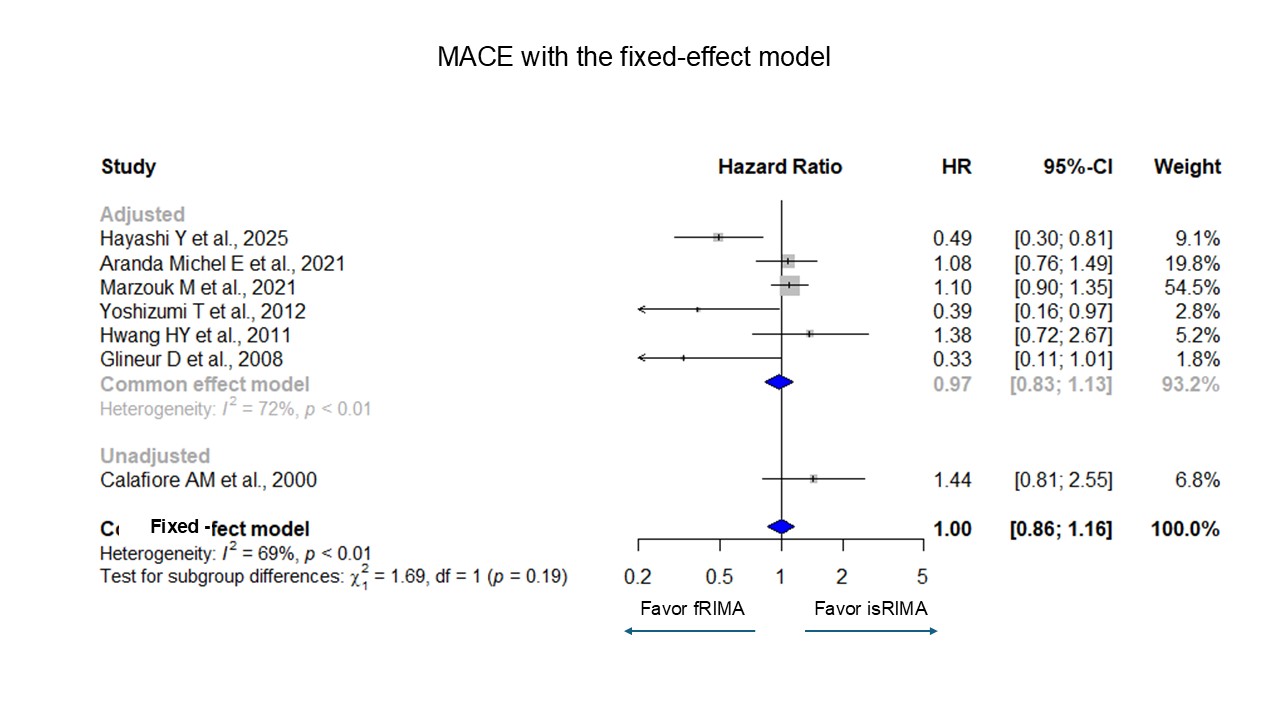

Supplement: ivag062_Supplementary_Data [file ivag062_supplementary_data.zip › 09-Mar-2026_111718_Revised_Supplemental_Figure_S5C..png.jpg]
