# Supplementary material for: Free Versus In situ Right Internal Mammary Artery as a Conduit in Coronary Artery Bypass Surgery: A Meta-Analysis
Source: Interdiscip Cardiovasc Thorac Surg. 2026 Feb 25;41(4):ivag062. doi: 10.1093/icvts/ivag062 (PMC13043274; doi:10.1093/icvts/ivag062)

Overall mortality leave-one-out

Study	HR	95%-CI	P-value	Tau2	Tau	I2
Hayashi Y et al. 2025	1.45	[1.14; 1.83]	< 0.01	0	0	0%
Aranda-Michel E et al. 2021	1.10	[0.72; 1.70]	0.66	0.1705	0.4129	50%
Marzouk M et al. 2020	1.06	[0.67; 1.66]	0.81	0.1631	0.4039	39%
Magruder JT et al. 2016 LC	1.10	[0.75; 1.62]	0.63	0.1358	0.3685	47%
Magruder JT et al. 2016 RC	1.12	[0.75; 1.67]	0.59	0.1542	0.3927	50%
Yoshizumi T et al. 2012	1.21	[0.84; 1.73]	0.31	0.1071	0.3273	42%
Hwang HY et al. 2011	1.14	[0.76; 1.72]	0.53	0.1616	0.4019	51%
Glineur D et al. 2008	1.17	[0.79; 1.74]	0.42	0.1453	0.3812	49%
Calafiore AM et al. 2000	1.10	[0.69; 1.73]	0.69	0.1937	0.4401	50%
Tashiro T et al. 1998	1.15	[0.74; 1.78]	0.52	0.1762	0.4198	51%
**Random effects model**	**1.15**	**[0.79; 1.68]**	**0.47**	**0.1355**	**0.3680**	**45%**

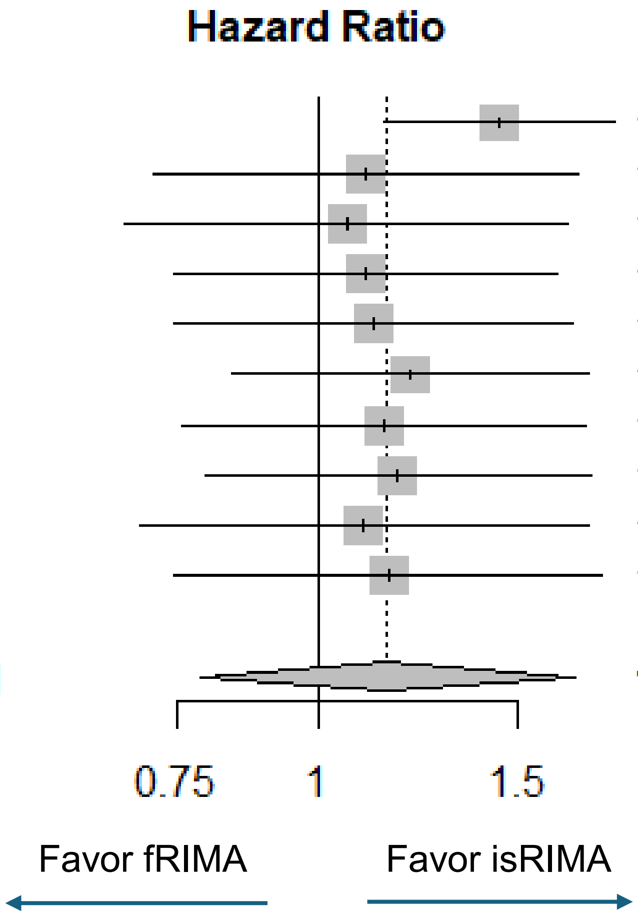

Supplement: ivag062_Supplementary_Data [file ivag062_supplementary_data.zip › Supplementary_Data.zip!/Supplemental Figure S4A.pdf]
