# Supplementary material for: Free Versus In situ Right Internal Mammary Artery as a Conduit in Coronary Artery Bypass Surgery: A Meta-Analysis
Source: Interdiscip Cardiovasc Thorac Surg. 2026 Feb 25;41(4):ivag062. doi: 10.1093/icvts/ivag062 (PMC13043274; doi:10.1093/icvts/ivag062)

Graft occlusion leave-one-out

Study	HR	95%-CI	P-value	Tau2	Tau	I2
Bakaeen FG et al. 2022 Ao inflow	1.04	[0.87; 1.24]	0.66	0.0199	0.1410	75%
Bakaeen FG et al. 2022 LIMA inflow	1.02	[0.87; 1.21]	0.80	0.0188	0.1372	75%
Bakaeen FG et al. 2022 SVG inflow	1.00	[0.94; 1.07]	0.98	0.0011	0.0336	15%
Isomura T et al. 2021	1.13	[0.80; 1.59]	0.50	0.1074	0.3278	73%
Yoshizumi T et al. 2012	1.04	[0.88; 1.22]	0.65	0.0179	0.1336	74%
Tatoulis J et al. 2011	1.07	[0.91; 1.27]	0.39	0.0167	0.1293	72%
Fukui T et al. 2010	1.10	[0.78; 1.54]	0.59	0.1014	0.3184	73%
**Random effects model**	**1.03**	**[0.88; 1.21]**	**0.72**	**0.0177**	**0.1331**	**71%**

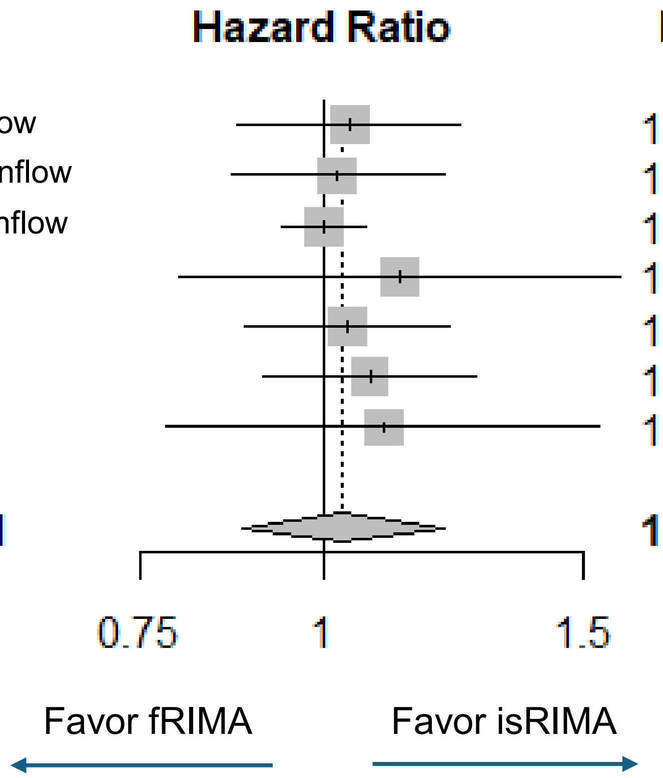

Supplement: ivag062_Supplementary_Data [file ivag062_supplementary_data.zip › Supplementary_Data.zip!/Supplemental Figure S4B.pdf]
