# Supplementary material for: Free Versus In situ Right Internal Mammary Artery as a Conduit in Coronary Artery Bypass Surgery: A Meta-Analysis
Source: Interdiscip Cardiovasc Thorac Surg. 2026 Feb 25;41(4):ivag062. doi: 10.1093/icvts/ivag062 (PMC13043274; doi:10.1093/icvts/ivag062)

MACE leave-one-out

Study	HR	95%-CI	P-value	Tau2	Tau	I2
Hayashi Y et al. 2025	1.00	[0.74; 1.35]	0.99	0.0625	0.2500	53%
Aranda-Michel E et al. 2021	0.80	[0.52; 1.25]	0.33	0.1988	0.4458	74%
Marzouk M et al. 2020	0.79	[0.50; 1.25]	0.31	0.2128	0.4613	71%
Yoshizumi T et al. 2012	0.94	[0.68; 1.31]	0.73	0.0955	0.3090	67%
Hwang HY et al. 2011	0.81	[0.56; 1.17]	0.26	0.1327	0.3643	72%
Glineur D et al. 2008	0.94	[0.68; 1.29]	0.69	0.0971	0.3115	67%
Calafiore AM et al. 2000	0.79	[0.55; 1.15]	0.23	0.1307	0.3616	71%
**Random effects model**	**0.87**	**[0.62; 1.21]**	**0.41**	**0.1188**	**0.3447**	**69%**

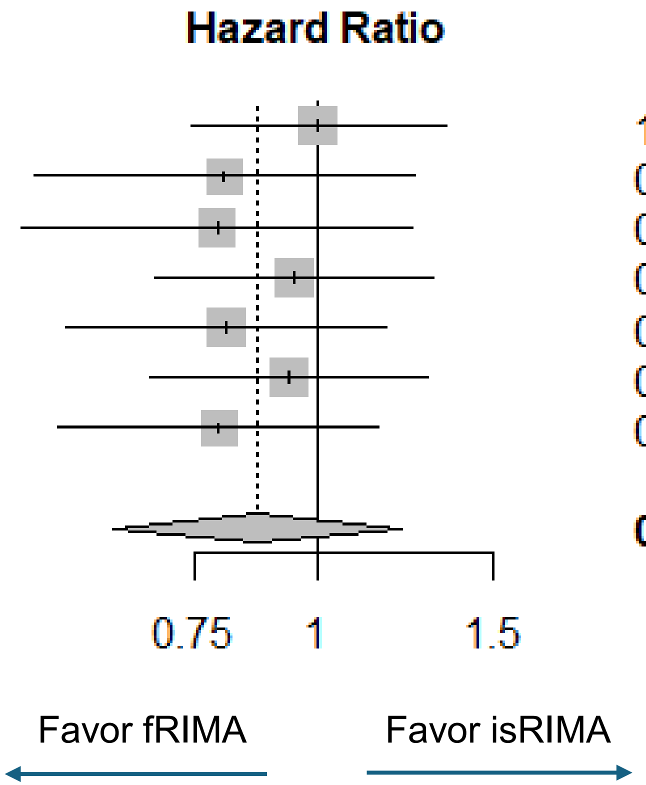

Supplement: ivag062_Supplementary_Data [file ivag062_supplementary_data.zip › Supplementary_Data.zip!/Supplemental Figure S4C.pdf]
