# Supplementary material for: Free Versus In situ Right Internal Mammary Artery as a Conduit in Coronary Artery Bypass Surgery: A Meta-Analysis
Source: Interdiscip Cardiovasc Thorac Surg. 2026 Feb 25;41(4):ivag062. doi: 10.1093/icvts/ivag062 (PMC13043274; doi:10.1093/icvts/ivag062)

Repeat revascularization leave-one-out

Study	HR	95%-CI	P-value	Tau2	Tau	I2
Hayashi Y et al. 2025	1.72	[0.94; 3.15]	0.08	0.2251	0.4744	49%
Marzouk M et al. 2020	1.38	[0.58; 3.27]	0.47	0.6576	0.8109	70%
Magruder JT et al. 2016 LC	1.08	[0.57; 2.07]	0.81	0.2763	0.5257	53%
Magruder JT et al. 2016 RC	1.14	[0.52; 2.49]	0.73	0.4999	0.7071	66%
Hwang HY et al. 2011	1.29	[0.53; 3.16]	0.57	0.7161	0.8462	72%
Glineur D et al. 2008	1.50	[0.73; 3.09]	0.27	0.4521	0.6724	68%
**Random effects model**	**1.34**	**[0.68; 2.66]**	**0.40**	**0.4482**	**0.6695**	**64%**

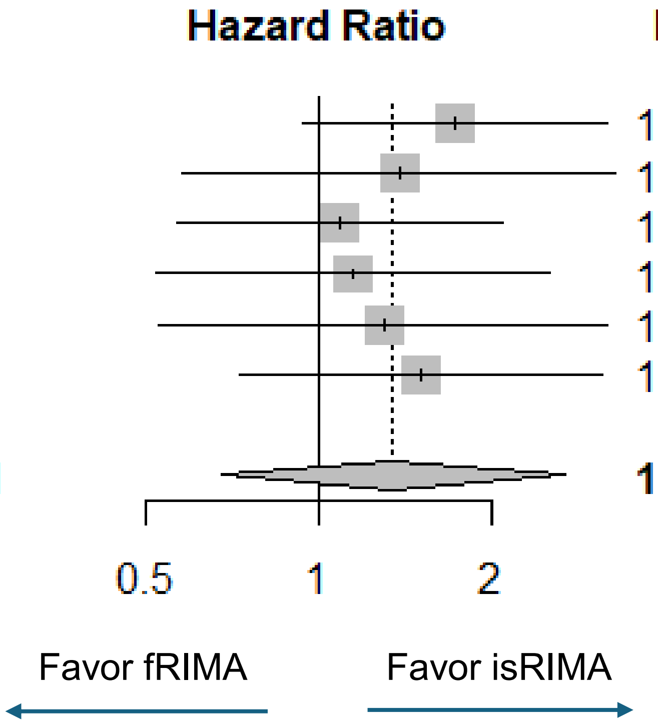

Supplement: ivag062_Supplementary_Data [file ivag062_supplementary_data.zip › Supplementary_Data.zip!/Supplemental Figure S4D.pdf]
