# Supplementary material for: Free Versus In situ Right Internal Mammary Artery as a Conduit in Coronary Artery Bypass Surgery: A Meta-Analysis
Source: Interdiscip Cardiovasc Thorac Surg. 2026 Feb 25;41(4):ivag062. doi: 10.1093/icvts/ivag062 (PMC13043274; doi:10.1093/icvts/ivag062)
Supplement: ivag062_Supplementary_Data [file ivag062_supplementary_data.zip › Supplementary_Data.zip!/Revised Supplemental Figure Legends ICVTS.docx]

**Supplemental Figure Legends**

**Supplemental Figure S1A.**

Risk of bias summary assessed with the RoB 2 tool.

Each row represents an individual randomized controlled trial, and each column represents a domain of bias according to the Cochrane Risk of Bias 2 (RoB 2) tool:
(D1) bias arising from the randomization process;
(D2) bias due to deviations from intended interventions;
(D3) bias due to missing outcome data;
(D4) bias in measurement of the outcome; and
(D5) bias in selection of the reported result.

Judgments are expressed as low risk of bias, some concerns, or high risk of bias, and the overall risk of bias for each study is shown in the rightmost column.

**Supplemental Figure S1B.**

Risk of bias summary assessed with the ROBINS-I tool.

Each row represents an individual non-randomized study of interventions, and each column represents a domain of bias according to the Risk Of Bias In Non-randomized Studies of Interventions (ROBINS-I) tool:
(D1) bias due to confounding;
(D2) bias in classification of interventions;
(D3) bias due to deviations from intended interventions;
(D4) bias due to missing data;
(D5) bias in measurement of outcomes; and
(D6) bias in selection of the reported result.

Risk of bias judgments are categorized as low, moderate, serious, or critical risk of bias. The overall risk of bias for each study is presented in the rightmost column.

**Supplemental Figures S2A-D.**

Publication bias assessment using Funnel plot with p-value of Egger’s regression test (only for overall mortality due to the number of studies) for S2A) Overall mortality, S2B) Graft occlusion, S2C) MACE, and S2D) Repeat revascularization. LC, left coronary territory target; RC, right coronary territory target; Aorta, aorta inflow; LIMA, left internal mammary artery composite inflow; SVG, saphenous vein graft composite inflow; MACE, major adverse cardiac events.

**Supplemental Figures S3A-B.**

The results of the sensitivity analysis by excluding studies requiring OR-to RR conversion for S3A) Graft occlusion and S3B) Repeat revascularization. fRIMA, free right internal mammary artery; isRIMA, in-situ right internal mammary artery; HR, hazard/relative/risk ratio, CI, confidence interval; LAD, left anterior descending coronary artery.

**Supplemental Figures S4A-D.**

The results of leave-one-out analyses for S4A) Overall mortality, S4B) Graft occlusion, S4C) MACE, and S4D) Repeat revascularization.

LC, left coronary territory target; RC, right coronary territory target; Aorta, aorta inflow; LIMA, left internal mammary artery composite inflow; SVG, saphenous vein graft composite inflow; HR, hazard ratio; CI, confidence interval; I2, heterogeneity.

**Supplemental Figures S5A-D.**

The results of the fixed-effect model for S5A) overall mortality, S5B) Graft occlusion, S5C) MACE, S5D) repeat revascularization.

MACE, major adverse cardiac event; HR, hazard/relative/risk ratio; CI, confidence interval.

**Supplemental Figures S6A and B.**

Bubble plot illustrating the results of the meta-regression analysis examining the association between the difference in preoperative prevalence of hypertension S6A) and preoperative prevalence of peripheral vascular disease S6B), and the effect size of repeat revascularization. Each bubble represents an individual study, with the size of the bubble proportional to the study’s weight in the analysis. The regression line indicates a positive relationship between the difference in preoperative prevalence of hypertension and peripheral vascular disease, and the effect size. HTN, hypertension; PVD, peripheral vascular disease.

**Supplemental Figures S7A and B.**

Forest plots for S7A) overall mortality and S7B) MACE with grouping adjusted (RCT, PSMs, and multivariate adjustment) and unadjusted, excluding the studies by Tashiro et al. (1998) and Calafiore et al. (2000). The horizontal lines represent the values within the 95% confidence interval of the underlying effects. The vertical line indicates a hazard or relative/risk ratio of 1. The vertical dot line indicates the mean of the total hazard and relative/risk ratios. CI, confidence interval; HR, hazard/relative/risk ratio; MACE, major adverse cardiac events.
