# Supplementary material for: Free Versus In situ Right Internal Mammary Artery as a Conduit in Coronary Artery Bypass Surgery: A Meta-Analysis
Source: Interdiscip Cardiovasc Thorac Surg. 2026 Feb 25;41(4):ivag062. doi: 10.1093/icvts/ivag062 (PMC13043274; doi:10.1093/icvts/ivag062)
Supplement: ivag062_Supplementary_Data [file ivag062_supplementary_data.zip › Supplementary_Data.zip!/Supplemental Figure S1B.pdf]

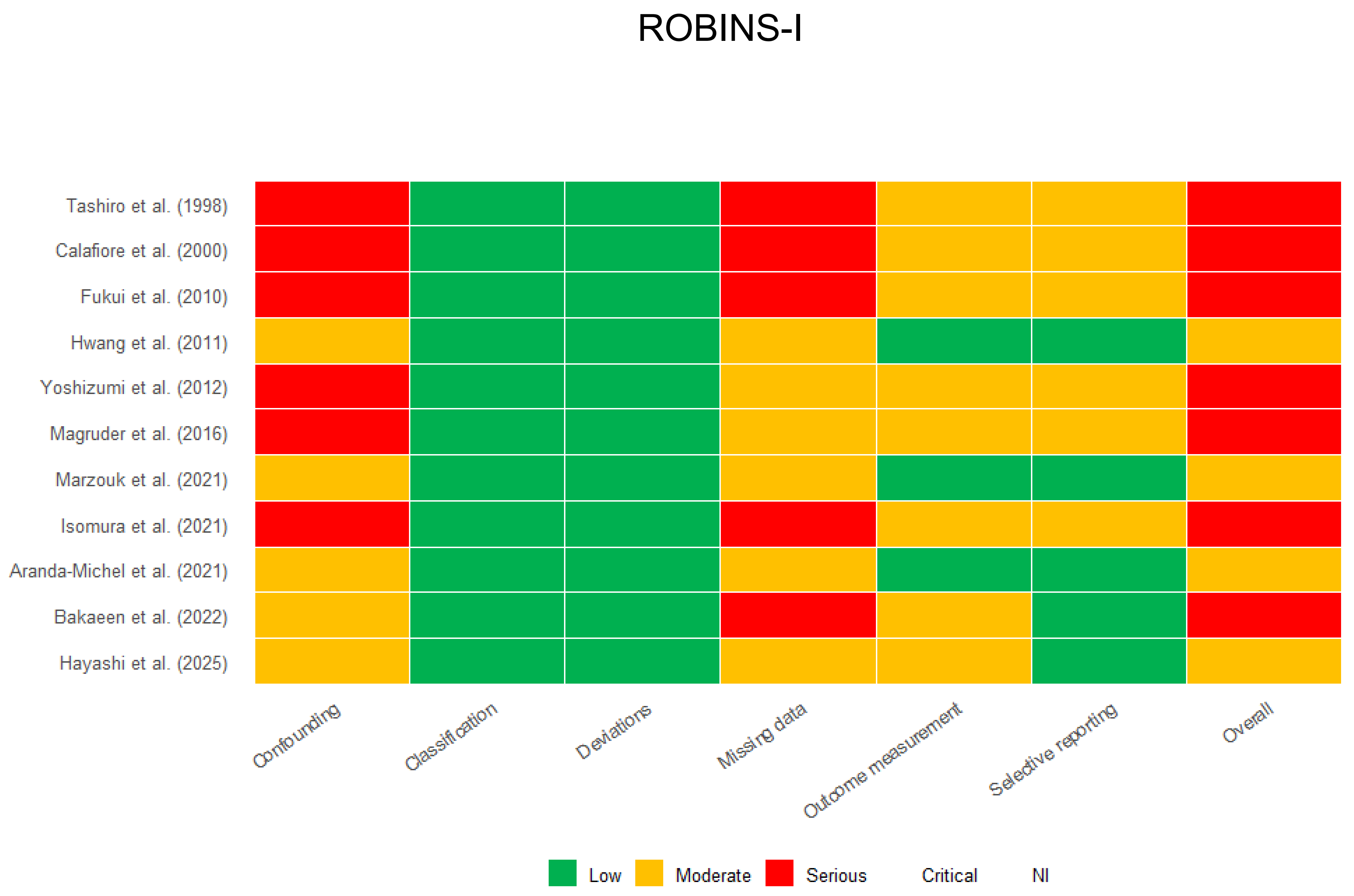
ROBINS-I
Tashiro et al. (1998)
Calafiore et al. (2000)
Fukui et al. (2010)
Hwang et al. (2011)
Yoshizumi et al. (2012)
Magruder et al. (2016)
Marzouk et al. (2021)
Isomura et al. (2021)
Aranda-Michel et al. (2021)
Bakaeen et al. (2022)
Hayashi et al. (2025)
Confounding
Classification
Deviations
Missing data
Outcome measurement
Selective reporting
Overall
Low
Moderate
Serious
Critical
NI
