# Supplementary material for: Free Versus In situ Right Internal Mammary Artery as a Conduit in Coronary Artery Bypass Surgery: A Meta-Analysis
Source: Interdiscip Cardiovasc Thorac Surg. 2026 Feb 25;41(4):ivag062. doi: 10.1093/icvts/ivag062 (PMC13043274; doi:10.1093/icvts/ivag062)
Supplement: ivag062_Supplementary_Data [file ivag062_supplementary_data.zip › Supplementary_Data.zip!/Supplemental Figure S2A.pdf]

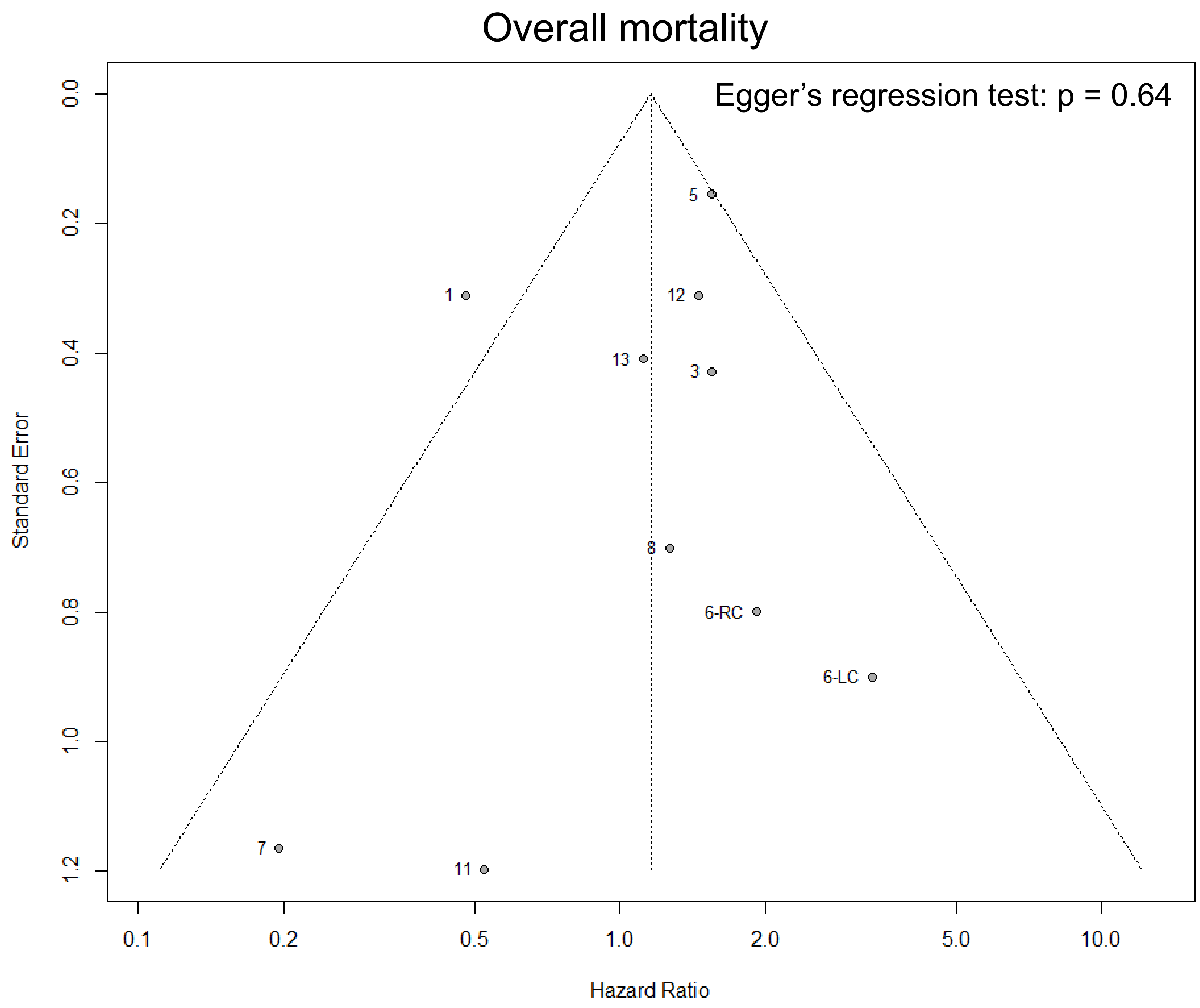
Overall mortality
Egger's regression test: p = 0.64
Standard Error
0.0
0.2
0.4
0.6
0.8
1.0
1.2
5
1
12
13
3
8
6-RC
6-LC
7
11
0.1
0.2
0.5
1.0
2.0
5.0
10.0
Hazard Ratio
