# Supplementary material for: Free Versus In situ Right Internal Mammary Artery as a Conduit in Coronary Artery Bypass Surgery: A Meta-Analysis
Source: Interdiscip Cardiovasc Thorac Surg. 2026 Feb 25;41(4):ivag062. doi: 10.1093/icvts/ivag062 (PMC13043274; doi:10.1093/icvts/ivag062)
Supplement: ivag062_Supplementary_Data [file ivag062_supplementary_data.zip › Supplementary_Data.zip!/Supplemental Figure S2C.pdf]

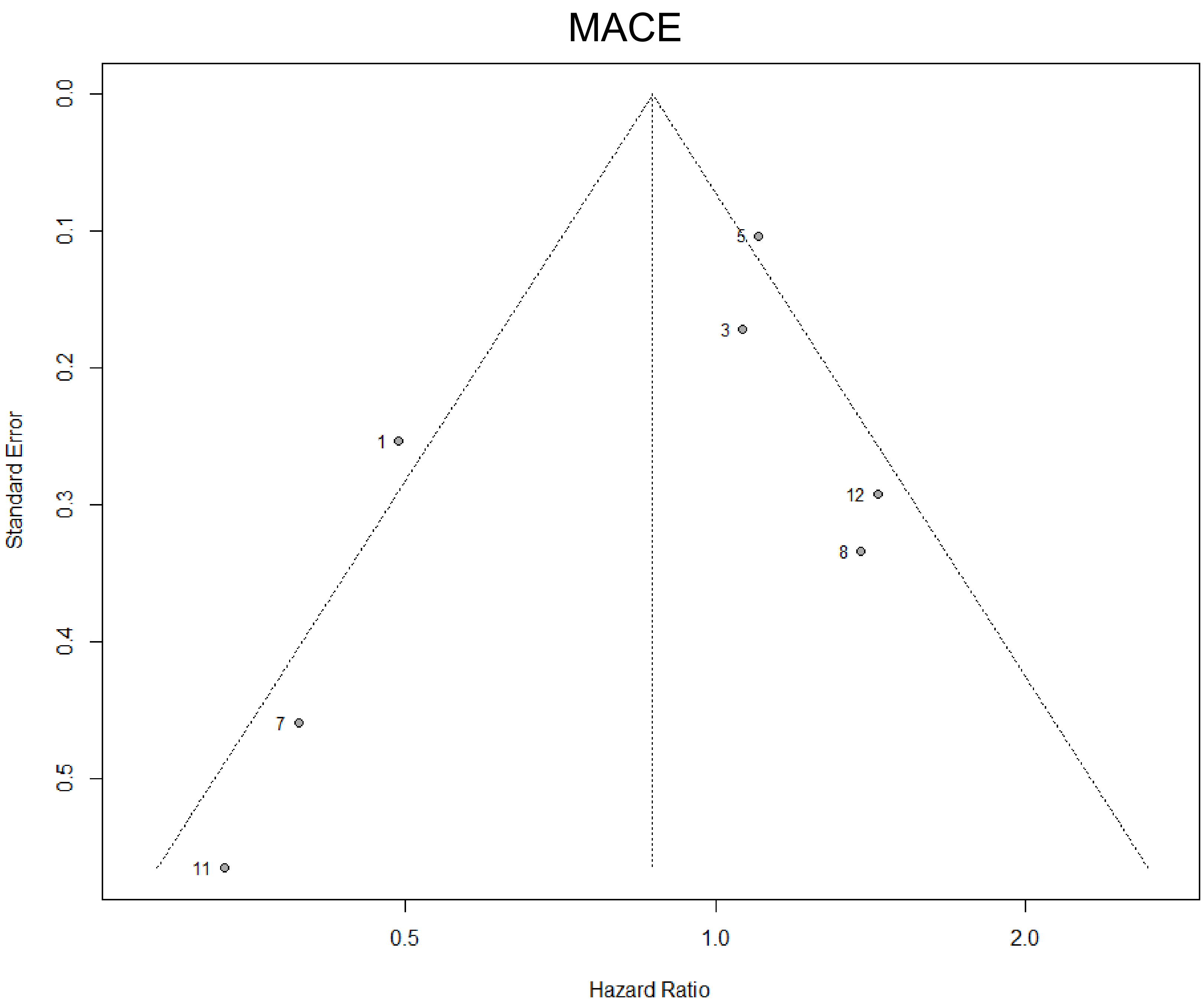

MACE
Standard Error
0.0
0.1
0.2
0.3
0.4
0.5
Hazard Ratio
0.5
1.0
2.0
1
3
5
7
8
11
12
